# Testing the role of predicted gene knockouts in human anthropometric trait variation

**DOI:** 10.1093/hmg/ddw055

**Published:** 2016-02-21

**Authors:** Samuel Lessard, Alisa K. Manning, Cécile Low-Kam, Paul L. Auer, Ayush Giri, Mariaelisa Graff, Claudia Schurmann, Hanieh Yaghootkar, Jian'an Luan, Tonu Esko, Tugce Karaderi, NHLBI GO Exome Sequence Project, GOT2D, T2D-GENES, GIANT Consortium, Erwin P. Bottinger, Yingchang Lu, Chris Carlson, Mark Caulfield, Marie-Pierre Dubé, Rebecca D. Jackson, Charles Kooperberg, Barbara McKnight, Ian Mongrain, Ulrike Peters, Alex P. Reiner, David Rhainds, Nona Sotoodehnia, Joel N. Hirschhorn, Robert A. Scott, Patricia B. Munroe, Timothy M. Frayling, Ruth J.F. Loos, Kari E. North, Todd L. Edwards, Jean-Claude Tardif, Cecilia M. Lindgren, Guillaume Lettre

**Affiliations:** ^1^Montreal Heart Institute, Montréal, Québec H1T 1C8, Canada; ^2^Faculté de Médecine, Université de Montréal, Montréal, Québec H3T 1J4, Canada; ^3^Medical and Population Genetics Program, Broad Institute, Cambridge, MA 02142, USA; ^4^Center for Human Genetics Research, Massachusetts General Hospital, Boston, MA 02114, USA; ^5^Department of Medicine and; ^6^Department of Genetics, Harvard Medical School, Boston, MA 02115, USA; ^7^School of Public Health, University of Wisconsin-Milwaukee, Milwaukee, WI 53201-0413, USA; ^8^Division of Epidemiology, Institute for Medicine and Public Health and; ^9^Department of Medicine, Vanderbilt Genetics Institute, Vanderbilt University Medical Center, Nashville, TN 37203, USA; ^10^University of North Carolina Gillings School of Global Public Health, Chapel Hill, NC 27599, USA; ^11^The Charles Bronfman Institute for Personalized Medicine and; ^12^The Mindich Child Health and Development Institute, the Genetics of Obesity and Related Metabolic Traits Program, The Icahn School of Medicine at Mount Sinai, New York, NY 10029, USA; ^13^Genetics of Complex Traits, University of Exeter Medical School, University of Exeter, Exeter EX2 5DW, UK; ^14^Medical Research Council Epidemiology Unit, University of Cambridge, Cambridge CB2 0QQ, UK; ^15^Estonian Genome Center, University of Tartu, Tartu, Estonia; ^16^Division of Endocrinology, Genetics and Basic and Translational Obesity Research, Children's Hospital Boston, Boston, MA 02115, USA; ^17^Wellcome Trust Centre for Human Genetics, University of Oxford, Oxford OX3 7BN, UK; ^18^Division of Public Health Sciences, Fred Hutchinson Cancer Research Center, Seattle, WA 98109-1024, USA; ^19^Clinical Pharmacology, William Harvey Research Institute and; ^20^NIHR Barts Cardiovascular Biomedical Research Unit, Barts and The London School of Medicine and Dentistry, Queen Mary University of London, London EC1M 6BQ, UK; ^21^Division of Endocrinology, Diabetes and Metabolism, Ohio State University, Columbus, OH 43210, USA; ^22^Department of Biostatistics, University of Washington, Seattle, WA 98195, USA; ^23^Division of Cardiology, Cardiovascular Health Research Unit, University of Washington, Seattle, WA 98195-6422, USA; ^24^The Big Data Institute, University of Oxford, Oxford, UK

## Abstract

Although the role of complete gene inactivation by two loss-of-function mutations inherited in *trans* is well-established in recessive Mendelian diseases, we have not yet explored how such gene knockouts (KOs) could influence complex human phenotypes. Here, we developed a statistical framework to test the association between gene KOs and quantitative human traits. Our method is flexible, publicly available, and compatible with common genotype format files (e.g. PLINK and vcf). We characterized gene KOs in 4498 participants from the NHLBI Exome Sequence Project (ESP) sequenced at high coverage (>100×), 1976 French Canadians from the Montreal Heart Institute Biobank sequenced at low coverage (5.7×), and >100 000 participants from the Genetic Investigation of ANthropometric Traits (GIANT) Consortium genotyped on an exome array. We tested associations between gene KOs and three anthropometric traits: body mass index (BMI), height and BMI-adjusted waist-to-hip ratio (WHR). Despite our large sample size and multiple datasets available, we could not detect robust associations between specific gene KOs and quantitative anthropometric traits. Our results highlight several limitations and challenges for future gene KO studies in humans, in particular when there is no prior knowledge on the phenotypes that might be affected by the tested gene KOs. They also suggest that gene KOs identified with current DNA sequencing methodologies probably do not strongly influence normal variation in BMI, height, and WHR in the general human population.

## Introduction

The identification of complete loss-of-function (LoF) alleles (i.e. genetic null or amorphic alleles) is a powerful strategy to characterize gene functions through random (e.g. chemical mutagenesis) or targeted [e.g. knockout (KO) methodology in the mouse, RNAi] genetic experiments. In contrast to model organisms, humans are not amenable to such genetic manipulations. Yet, there is tremendous biomedical interest in understanding how the complete disruption of both copies of a gene may impact human biology (1). Our complex physiology, interactions with our environment, and gene redundancy within our genome are only few of the reasons highlighting the importance of describing the phenotypic consequences of gene inactivation in humans. From a drug development perspective, the identification of humans with gene KOs also offers naturally occurring genetic experiments to assess the potential pleiotropic effects of candidate target genes (2).

Mendelian diseases, such as sickle cell anemia [MIM 603903] and cystic fibrosis [MIM 219700], offer an entry point into the study of gene functions in humans. Indeed, the study of these conditions continues to yield important insights into human biology in health and disease (3). But only a limited number of genes have been implicated in Mendelian diseases: as of October 13, 2015, there were 4651 genes in the Online Mendelian Inheritance in Man (OMIM) database with phenotype-causing mutations. Furthermore, these mutations are often rare such that it is difficult to assemble sufficiently large cohorts of patients to study their pleiotropic effects. Gene KOs can have strong phenotypic effects on anthropometric traits in the context of Mendelian disorders or syndromes, as evident by mutations causing early-onset morbid obesity (*PCSK1*, *LEPR*) or dwarfism (*GH1 GHR, ATR*) (4–6). These mutations are rare (often private) and unlikely to be involved in anthropometric trait variation in the general population. However, the possibility that gene KOs of more subtle effect might influence normal variation in anthropometric traits remains to be investigated.

Large-scale whole-exome and -genome sequencing projects are beginning to systematically catalogue coding genetic variation in the human genome, including predicted LoF variants (7–11). On average, there are ∼100–200 LoF variants per individual, resulting in ∼20 genes that are inactivated through homozygosity or compound heterozygosity (12). These numbers include mostly common variants, which are more likely to be phenotypically neutral given the effect of purifying selection (13). Limiting to variants with a minor allele frequency (MAF) <0.5%, the 1000 Genomes Project estimated that there are 10–20 LoF variants per individual (8). LoF variants are usually defined as variants that truncate protein sequences [nonsense and frameshift insertion-deletion (indel)] or that abrogate splice sites or stop codons (stop-loss) (12). Using this definition of LoF variant, and limiting their analyses to variants with a MAF < 2%, Sulem *et al*. found that ∼8% of 104 220 Icelanders carry at least one complete gene KO, and that most gene KOs are seen in <5 individuals (14).

Recently, several studies have explored the link between gene KOs and human complex phenotypes, such as chronic diseases (12,15–17) and autism (18). As mentioned above, it is well-established that rare gene inactivation can cause extreme anthropometric phenotypes in several human recessive disorders. The goal of our study is to extend this observation and determine whether gene KOs of modest phenotypic effect also contribute to anthropometric trait variation in the general human population. We developed a statistical method to test for association between predicted gene KOs and quantitative human phenotypes and characterized the distribution of predicted gene KOs in 2772 European Americans and 1726 African Americans from the National Heart, Lung, and Blood Institute (NHLBI) Exome Sequence Project (ESP). We then applied our method to detect associations between gene KOs and three quantitative anthropometric traits [body mass index (BMI), adult height, and BMI-adjusted waist-to-hip ratio (WHR)] using high coverage whole-exome sequence (WES) data from 4498 ESP participants, low coverage whole-genome sequence (WGS) data from 1969 French Canadians, and >100 000 participants from the Genetic Investigation of ANthropometric Traits (GIANT) Consortium genotyped on an exome array.

## Results

### Number and distribution of predicted gene KOs in ESP

We identified 18 137 and 21 935 LoF variants in 1726 African Americans and 2772 European Americans from ESP, respectively (Table 1 and Supplementary Material, Table S1). These LoF variants included protein truncating (nonsense, frameshift indel), stop-loss and splice site variants. On average, we found 65 and 39 rare or low-frequency LoF variants (MAF < 5%) per African-American and European-American ESP participant, respectively (Table 1). These numbers are higher than some of the previous estimates (12,16,18), mostly because we included frameshift indels in our analyses. When excluding frameshift indels, we found on average 26 and 16 LoF variants with MAF < 5% per ESP African American and European American, respectively. Descriptive statistics on the number of LoF variants in ESP after excluding frameshift indels are available in Supplementary Material, Table S2. We screened the ESP dataset for individuals who are homozygous or compound heterozygous for LoF variants, and are therefore predicted KOs for a given gene. To detect compound heterozygosity, we used phased genotype information generated with the software Beagle to distinguish between LoF variants inherited in *cis* or *trans* (Table 1) (19). The identification of LoF variants depends on the gene annotation used. To address this concern, we re-analyzed the ESP WES data using the GENCODE basic transcripts annotation instead of RefSeq, and only considered variants that fell within all transcripts for a given gene. We obtained very similar association results between the two annotation software (Supplementary Material, Fig. S1). We present below results generated with the RefSeq annotation.

**Table 1.** Number and frequency of predicted gene knockouts (KO) in 1727 African Americans and 2772 European Americans from the NHLBI Exome Sequence Project (ESP)

			Variants/individuals	Variants/gene	Not phased		Phased	
					Gene KOs/individuals	Number of KO genes	Gene KOs/individuals	Number of KO genes
African Americans	All LoF (*N* = 18 137)		237	0.92	33.7	2530	25.9	2429
		Homozygotes			23.2	2384	23.2	2384
		Compound heterozygotes			10.4	601	2.6	334
	Rare LoF (*N* = 17 446)		65	0.89	4.2	2174	2.5	2071
		Homozygotes			2.3	2028	2.3	2028
		Compound heterozygotes			1.9	381	0.2	155
European Americans	All LoF (*N* = 21 935)		197	1.12	28.8	1844	23.2	1741
		Homozygotes			21.3	1694	21.3	1694
		Compound heterozygotes			7.6	487	1.9	247
	Rare LoF (*N* = 21 351)		39	1.09	1.8	1538	1.1	1433
		Homozygotes			1	1390	1.01	1390
		Compound heterozygotes			0.8	318	0.09	124

For this loss-of-function (LoF) variant analysis, we consider autosomal nonsense, stop-loss and splice site variants, as well as frameshift insertion-deletions (indels). Rare LoF variants have a minor allele frequency <5%. In the absence of phasing information, we assume that rare LoF are inherited in trans. As expected, considering phased genotype information significantly impacts the number of gene KOs that we can detect due to compound heterozygosity.

**Figure 1.**
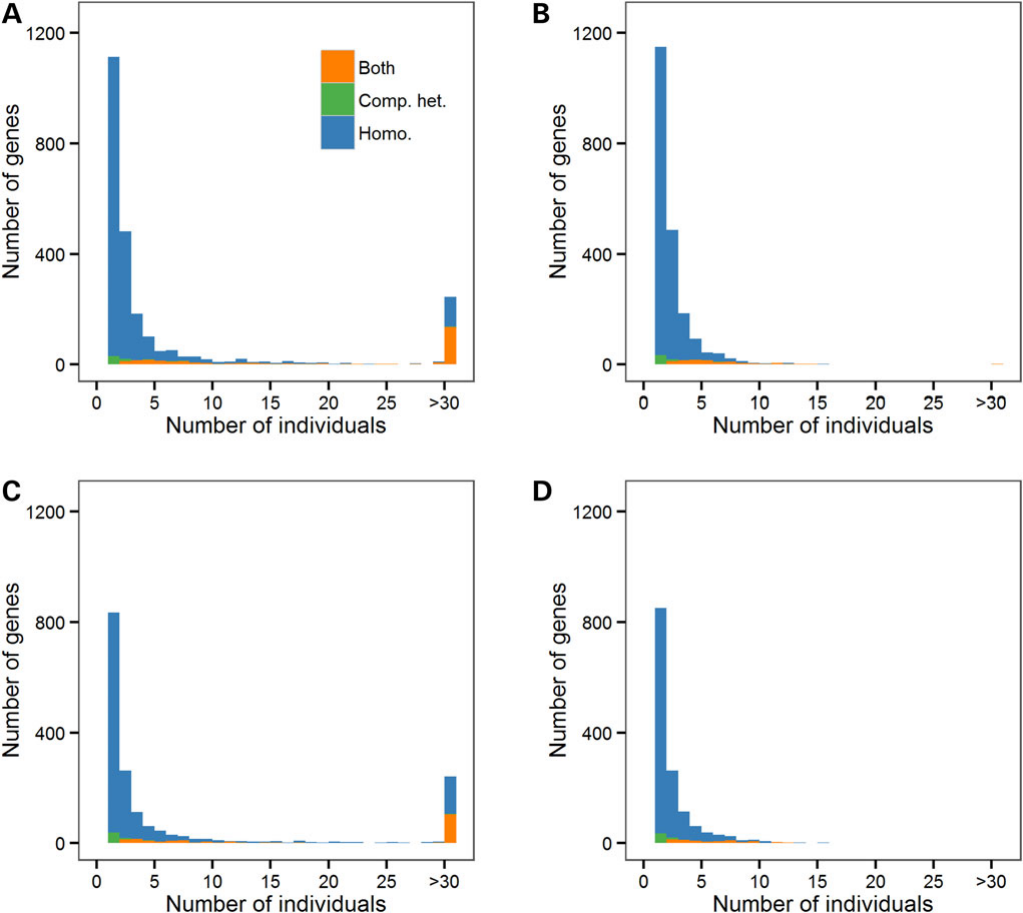
Distributions of the number of NHLBI Exome Sequence Project (ESP) participants with predicted gene knockouts (KOs). We present distributions in African Americans (**A** and **B**) and European Americans (**C** and **D**). We include all loss-of-function (LoF: nonsense, stop-loss, splice site, frameshift indel) variants in (A) and (C), whereas only rare/low-frequency LoF variants (minor allele frequency <5%) are included in (B) and (D). Homo., gene KO due to homozygosity; Comp. het., gene KO due to compound heterozygosity; Both, genes with homozygous and compound heterozygous LoF variants.

Common LoF variants are responsible for most predicted gene KOs (Fig. 1, and Supplementary Material, Fig. S2 for distributions without frameshift indels). For instance, in ESP African Americans, we found on average 25.9 and 2.5 predicted gene KOs per individual when analyzing all or rare/low-frequency LoF variants, respectively (Table 1). The corresponding numbers in European Americans are 23.2 and 1.1 for all and rare/low-frequency LoF variants (Table 1). While this article was under review, the Exome Aggregation Consortium (ExAC) reported an average of 35 homozygous protein-truncating variants per individual. This number is higher than the average number of homozygous LoF variants that we found in ESP (∼21–23/participant, Table 1) (20). This difference might simply reflect increased power in ExAC to discover rare mutations owing to its larger sample size (*N* = 60 706 versus *N* = 4498 for ESP). Because common LoF are more likely to be phenotypically neutral (13), we focused all subsequent analyses on LoF with MAF < 5% within ethnic group or sub-study. In the ESP dataset, we found 2071 and 1433 genes with both alleles inactivated by such LoF variants in at least one African American or one European American, respectively (Table 1). The higher number of predicted gene KOs in African Americans has been previously observed and is consistent with increased genetic diversity in African-ancestry populations (12). Overall, very few individuals shared the same gene KOs, most of them being found in only one individual (Fig. 1). Homozygosity of LoF variants is responsible for the majority of these KO events as we only found (after taking phase information into account) compound heterozygous individuals for ∼8% of the genes with at least one gene KO (Table 1). Stop-loss variants might not be as detrimental as other categories of LoF variants, but they are implicated in <0.9% of all gene KOs identified in ESP.

### Predicted gene KO associated with anthropometric traits in ESP

**Figure 2.**
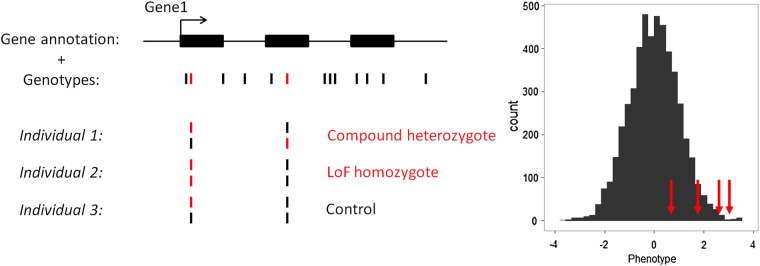
Schematic representation of the method to detect association between gene knockouts (KOs) and human quantitative variation. This example depicts a fictive gene with three exons (*GENE1*) that contains several SNPs. Our analytical framework only considers loss-of-function (LoF) variants (shown in red). *GENE1* KOs are individuals who are either compound heterozygous of homozygous for LoF variants (individual 1 and 2). The histogram shows the distribution of a normalized human quantitative trait. Our method tests whether individuals that are KOs for a given gene (red arrows) have on average more extreme phenotypes than the rest of the individuals.

**Figure 3.**
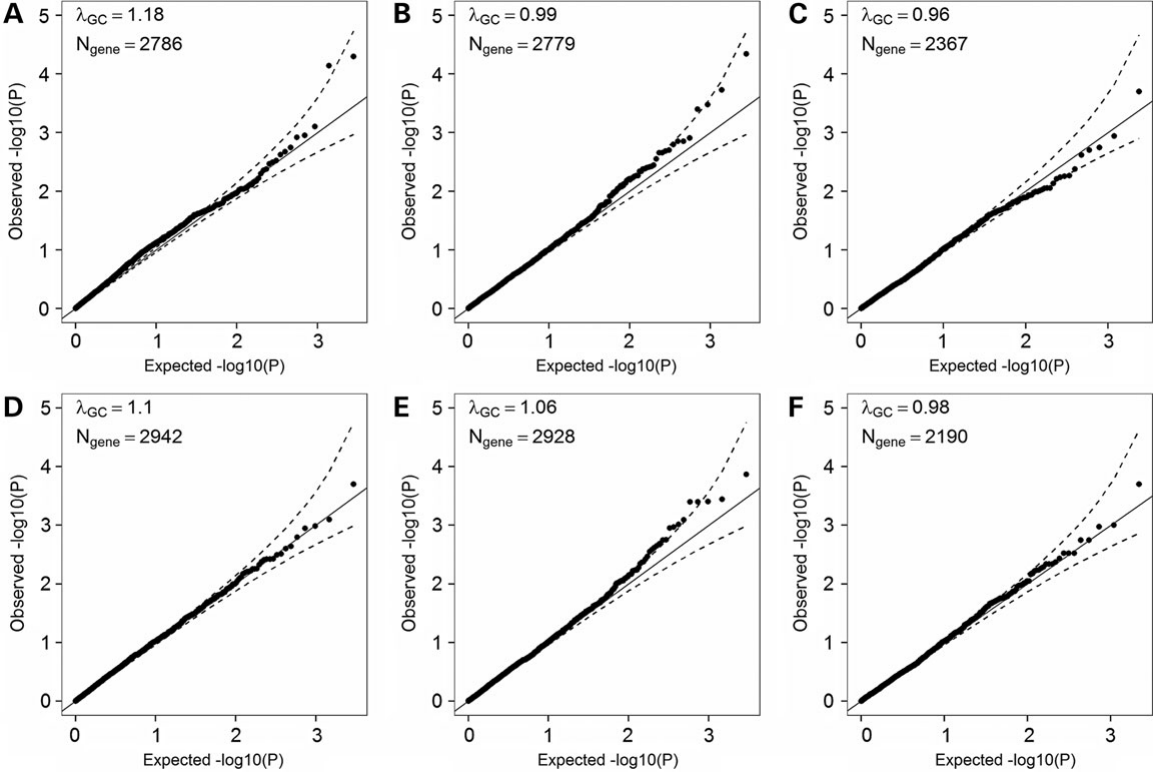
Quantile-quantile (QQ) plots of association results between predicted gene knockouts (KOs) and anthropometric traits in the (**A–C**) NHLBI Exome Sequence Project (ESP) and (**D–F**) GIANT ExomeChip datasets. In these datasets, we only considered loss of function (LoF) variants (nonsense, stop-loss, splice site, frameshift indels (ESP only)) with a minor allele frequency (MAF) <5%. We analyzed three anthropometric traits: (A) body mass index (BMI) (*N*_participants_ = 4475), (B) height (*N*_participants_ = 4423) and (C) waist-to-hip ratio (WHR) (*N*_participants_ = 2973). We performed these analyses stratified by ethnicity, and then combined the European American and African American results using meta-analysis methodology. We analyzed the same traits in the GIANT dataset: (D) BMI (*N*_participants_ = 103 838), (E) height (*N*_participants_ = 102 775) and (F) WHR (*N*_participants_ = 62 355). Results are not corrected for the genomic inflation factor. The dash lines correspond to the 95% confidence interval. *λ*_GC_, genomic inflation factor; *N*_gene_, number of genes with at least one participant that carries two LoF alleles.

We tested our newly developed method (Fig. 2) on three anthropometric traits (BMI, height, and WHR) that are available in a large number of ESP participants. We stratified our analyses by ethnicity and meta-analyzed association results (Fig. 3). Assuming that most genes are independent and given the number of genes for which we could find at least one predicted knocked out individual, we used the following Bonferroni-corrected significance threshold to declare significance: *α* = 2 × 10^−5^. No single genes reached this significance threshold for any of the three tested anthropometric traits after meta-analysis (Supplementary Material, Table S3).

To increase statistical power, we attempted to replicate genes with a nominal *P* < 0.05 in the ESP dataset using the WGS data from the Montreal Heart Institute (MHI) Biobank (*N* = 1976). We limited our analysis to genes with at least two KO individuals. Although the MHI Biobank dataset results from low-pass WGS, the number of identified LoF variants and gene KOs was similar to the number observed in ESP (Supplementary Material, Table S1), suggesting that the data are sufficiently comprehensive to support these analyses. We found that 30–40% of gene KOs in ESP were also knocked out in the MHI Biobank, highlighting the challenge to replicate such studies in humans. This might particularly be true for gene KOs observed only in ESP African Americans given that the MHI Biobank includes individuals of European ancestry. We combined the ESP and MHI Biobank results but we did not observe any significant associations with quantitative anthropometric traits (Supplementary Material, Table S3). We report results with a meta-analysis *P* < 0.005 in Table 2. The most promising gene KO association that we found is between *PKHD1L1* and lower BMI: we found 20 KO individuals for this gene who have on average a BMI that is 0.8 standard deviation (SD) below the population mean (corresponding to ∼−3.6 kg/m^2^). *PKHD1L1* may play a role in immunity (21).

**Table 2.** Association of gene knockouts (KOs) with anthropometric traits in the Exome Sequence Project (ESP) and Montreal Heart Institute (MHI) Biobank DNA sequencing datasets

Trait	Gene	ESP					MHI			Combined	
		Mean EA (real units)	*N*_KO_ EA	Mean AA (real units)	*N*_KO_ AA	*P*	Mean (real units)	*N*_KO_	*P*	Weighted average (real units)	*P*
BMI	*PKHD1L1*	0.7 (+3.2 kg/m^2^)	11	0.5 (+2.3 kg/m^2^)	6	0.009	1.6 (+7.2 kg/m^2^)	3	0.009	0.8 (+3.6 kg/m^2^)	0.0002
	*PLIN4*	2.7 (+12.2 kg/m^2^)	1	3.1 (+14.0 kg/m^2^)	1	5 × 10^−5^	−0.2 (−0.9 kg/m^2^)	2	0.67	1.4 (+6.3 kg/m^2^)	0.002
Height	*RMDN2*	NA	0	−1.1 (−7.0 cm)	4	0.03	−1.6 (−10.2 cm)	2	0.02	−1.3 (−8.3 cm)	0.002
	*ASIC4*	3.6 (23.0 cm)	1	1.5 (9.6 cm)	2	5 × 10^−5^	−0.4 (−2.6 cm)	2	0.56	1.2 (+7.7 cm)	0.002
	*SH2B2*	−1.6 (−10.2 cm)	2	NA	0	0.02	−1.9 (−12.2 cm)	1	0.06	−1.7 (−10.9 cm)	0.003
WHR	*C1QTNF5*	0.6 (+0.04)	1	1.8 (+0.13)	2	0.04	1.5 (+0.11)	2	0.03	1.4 (0.10)	0.003

We attempted to replicate gene KO associations from the ESP whole-exome DNA sequencing dataset in the MHI Biobank whole-genome DNA sequencing dataset. We tested for replication genes with *P* < 0.05 and at least two KO individuals in the ESP dataset. We report genes with a combined *P* < 0.005. We provide the mean gene KO effect size in standard deviation (SD) and metric units, assuming that 1 SD corresponds to 4.5 kg/m^2^, 6.4 cm, and 0.07 for BMI, height, and WHR respectively. *N*_KO_: number of individuals that are KO for the given gene.

EA: European-ancestry; AA: African-ancestry.

While examining the top candidate genes, we noticed that *PKHD1L1* is a large gene (78 exons, coding sequence is ∼14 kilobases), raising the possibility that our method could favor longer genes. In the ESP dataset, we found, as expected, that the number of LoF variants in a given gene is strongly correlated with the length of the coding sequence or the number of exons (all *P* < 1 × 10^−67^). However, the number of individuals who carry a rare gene KO is not correlated with the length of the coding sequence or the number of exons of the gene (all *P* > 0.2), except for a weak correlation observed in ESP African Americans with the length of the coding sequence (Pearson's *r* = 0.066, *P* = 0.003). To exclude the possibility that gene length may influence our results, we tested correlations with association results from the ESP and MHI Biobank combined analyses. With one exception (among 12 correlation tests performed), we found no significant correlations between the length of the coding sequence or the number of exons and association *P*-values for BMI, height, and WHR (all *P* > 0.25). In ESP African Americans, there was a weak correlation between the length of the coding sequence and the BMI *P*-values (Pearson's *r* = 0.069, *P* = 0.002), but it was in the opposite direction from our expectations (shorter genes have slightly more significant *P*-values). Together, these analyses suggest that our method to test association between gene KOs and human quantitative traits is largely insensitive to gene length.

### Gene KO identification and association testing using exome array data

Recognizing that the main limitation of our analysis is sample size, we contacted studies that are involved in the GIANT Consortium. Although WES or WGS data are not readily available for most of these studies, they all have genotyped their participants using an exome array that targets 250 000—mostly coding—variants. We reasoned that the large sample size offered by the GIANT Consortium could compensate for the limited number of variants present on the exome array. We recruited 22 studies, totaling >102 000 individuals (BMI and height available for all, WHR available for >62 000 individuals). Each study ran the method locally, stratifying all analyses by ethnicity, and we then combined results using meta-analysis methodology (22). The frequency of KO events was similar in ESP and the GIANT studies. However, there were more singletons (genes with a single KO individual) observed in European-ancestry individuals from the GIANT studies because of the very large sample size (Supplementary Material, Fig. S3).

We present the BMI, height, and WHR meta-analysis results for the GIANT studies in Figure 3. As reported above for the WES sequence datasets, and despite a sample size that is >10 times larger, we could not detect significant associations between gene KOs and quantitative anthropometric traits after accounting for the number of tests performed (Table 3 and Supplementary Material, Table S4). The most interesting finding pertains to the association between height and inactivation of *GRHPH*: autosomal recessive Mendelian mutations in this gene cause primary hyperoxaluria type 2 [MIM 260000] (23). Primary hyperoxaluria type 1 [MIM 259900], a more severe form of the disease caused by mutations in *AGXT*, is characterized by very severe growth failure (24). However, the connection between primary hyperoxaluria type 2 caused by recessive mutations in *GRHPH* and growth in humans has not been as clearly documented, although there is one case report of a child with this disease and short stature (25).

**Table 3.** Top association results between anthropometric traits and predicted gene knockouts (KOs) identified using ExomeChip data from 22 studies participating in the GIANT Consortium

Trait	Gene	*N*_KO_	*N*_study_	Weighted mean (SD)	*P*
BMI	*CYP20A1*	100	15	−0.35	0.001
	*ME2*	2	2	−1.90	0.002
	*KIAA1024*	7	5	−0.75	0.002
	*TBC1D5*	4	3	1.05	0.003
	*LRRC39*	147	16	0.23	0.003
	*TAS1R1*	191	6	0.15	0.004
	*LAMA3*	9	2	−1.02	0.004
	*KIAA0391*	3	2	−1.64	0.004
	*TAS2R60*	2	2	−2.04	0.005
Height	*GRHPR*	2	2	−2.28	0.0001
	*ABCB7*	365	10	−0.12	0.0003
	*ZDHHC14*	3	2	−2.01	0.0003
	*ZFPM1*	21	3	−0.60	0.0008
	*DHX57*	2	2	−2.04	0.0009
	*CD8A*	2	2	2.35	0.001
	*CDC42BPA*	4	2	1.70	0.001
	*NSUN4*	13	3	−0.78	0.002
	*ARPC5L*	6	2	−1.25	0.002
	*CCDC125*	45	9	0.35	0.002
	*BOK*	27	4	0.61	0.003
	*NSRP1*	9	1	−1.00	0.003
	*TEX13A*	2	1	2.00	0.004
	*RPGRIP1*	10	4	−0.72	0.004
	*SCGN*	6	5	−0.96	0.005
WHR	*C18orf56*	7	1	1.39	0.0002
	*AARS2*	3	2	−1.78	0.001
	*C18orf34*	6	3	1.27	0.002
	*CCDC68*	13	1	0.83	0.002
	*HRG*	3	2	−1.52	0.004
	*SPTA1*	2	2	1.86	0.004
	*SPTBN5*	191	11	0.15	0.005

We only report genes with *P* < 0.005 and at least two KO individuals. The weighted mean corresponds to the average phenotype (in standard deviation units) of individuals that are KO for this gene. *N*_KO_: number of individuals with a KO gene; *N*_study_: number of studies with at least one KO individual for a given gene.

### Prioritizing gene KOs using a candidate-gene approach

**Figure 4.**
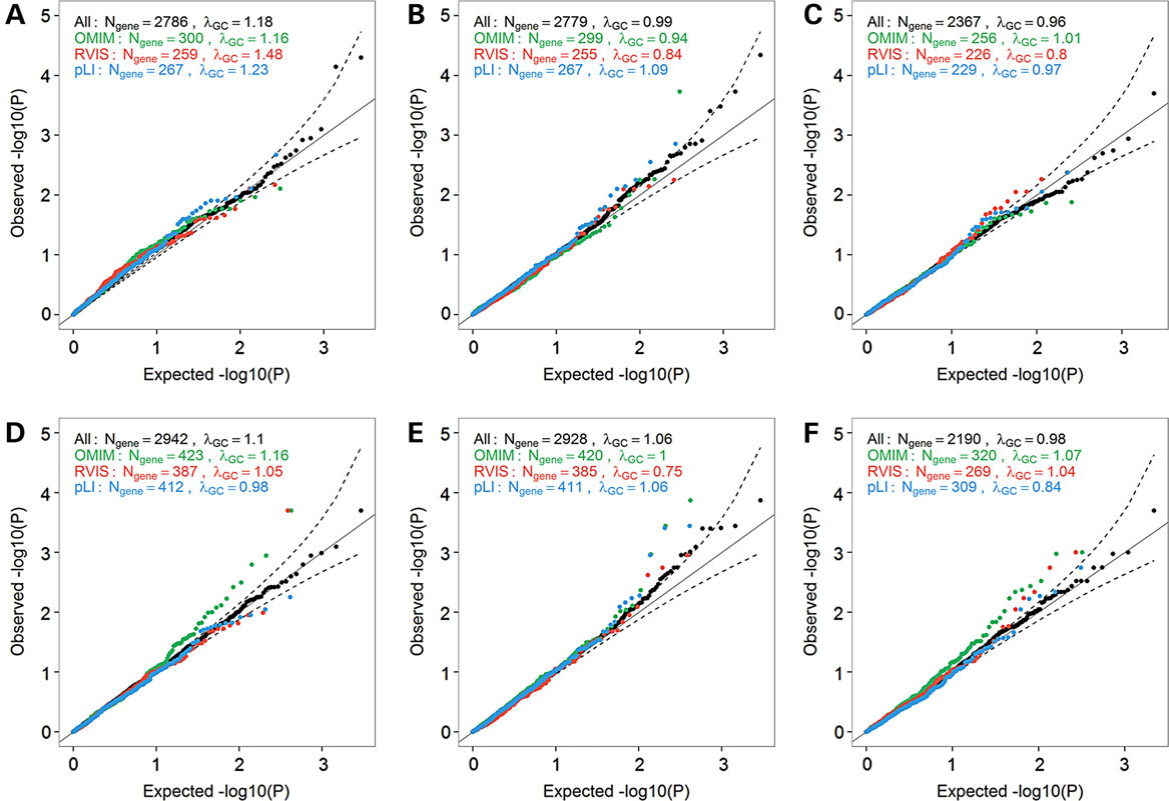
Quantile-quantile (QQ) plots of association results between predicted gene knockouts (KOs) in candidate-genes and anthropometric traits. We restricted these analyses to OMIM disease-causing genes (green), genes with Residual Variation Intolerance Score (RVIS) score <15% of RVIS scores for all genes in the human genome (red), or genes with a probability of being loss-of-function intolerant (pLI) score >0.9 (blue). We report results for three anthropometric traits in the NHLBI Exome Sequence Project (ESP): (**A**) body mass index (BMI) (*N*_participants_ = 4475), (**B**) height (*N*_participants_ = 4423) and (**C**) waist-to-hip ratio (WHR) (*N*_participants_ = 2973). We also report results for the same traits in the GIANT ExomeChip datasets: (**D**) BMI (*N*_participants_ = 103 838), (**E**) height (*N*_participants_ = 102 775), and (**F**) WHR (*N*_participants_ = 62 355). Results are not corrected for the genomic inflation factor. The dash lines correspond to the 95% confidence interval. *λ*_GC_, genomic inflation factor; *N*_gene_, number of genes with at least one participant that carries two LoF alleles.

We next asked whether we would increase power to detect associations between gene KO and anthropometric traits by restricting our analyses to strong candidate genes. We focused on subsets of genes that are associated with any phenotypes in OMIM, or genes that are intolerant to LoF mutations based on the Residual Variation Intolerance Score (RVIS) or the probability of being LoF Intolerant (pLI) score (20,26). We observed several genes that deviate from the null when restricting our analyses to these candidate genes, especially for the OMIM genes in the larger GIANT datasets for BMI and WHR (Fig. 4). We also reasoned that the Mouse Genome Informatics (MGI) database might be a good source of candidate genes for our human KO experiment. We retrieved the human homologues of genes from 30 MGI phenotype categories, and tested them against anthropometric traits (Supplementary Material, Fig. S5). Again, we observed inflation of the KO association results when compared to the null distribution, suggesting that some of these genes might influence anthropometric traits when completely inactivated. The most noticeable result was the distribution of test statistics in the GIANT BMI analysis for genes related to taste and olfaction (Supplementary Material, Fig. S5). Genes related to this category were significantly enriched for genes with a BMI *P* < 0.05 in GIANT (Fisher's exact test, *P* = 0.008).

## Discussion

We developed a simple statistical method to test the association between predicted gene KOs and human quantitative traits. We tested our method on three quantitative anthropometric traits (BMI, height, and WHR) in large DNA sequencing (ESP and MHI Biobank, >6400 individuals) and genotyping (22 participating GIANT studies, >102 000 individuals) datasets. Despite this large sample size, we did not identify significant genetic associations with predicted gene KOs, although the association between *PKHD1L1* and BMI or *GRHPH* and height are interesting and should be tested for replication. Within the limitations of our study design (sample size, incomplete catalogue of LoF variants), our results suggest that there are no predicted gene KOs with modest-to-large effect size on anthropometric trait variation in the general population. This conclusion is largely consistent with results from a recent study of homozygous LoF variants in 1432 individuals (17).

Importantly, our approach and results can guide future gene KO studies in humans. First, our method assumes that all LoF alleles for a given gene will shift the phenotypic mean in the same direction. Although it is a sensitive approach in this first large-scale gene KO experiment for quantitative traits, alternative methods could explore effect on phenotypic variance. Second, in order to maximize our sample size, we combined datasets from different technologies (WES, WGS, exome array). Although we accounted for this technical heterogeneity—gene KO statistics were similar across datasets—this approach could have introduced unanticipated biases. Ideally, high coverage WGS data would be available for gene KO studies. Third, haplotype phasing of DNA sequence data from unrelated individuals (ESP and MHI Biobank), and the lack of phase information for the GIANT ExomeChip studies, has limited our ability to identify compound heterozygous individuals. This could impact our results as nearly 20% of all gene KOs identified in this study were due to compound heterozygosity. We note, however, that excluding compound heterozygotes from the ESP analyses had very limited impact on our results (Supplementary Material, Fig. S6). Fourth, we only considered nonsense, splice site, stop-loss and frameshift indels as LoF variants to identify gene KOs. Some of these variants are likely neutral: for instance, genes are more tolerant to non-synonymous variants at the 3′ end of a gene, and nearby variants can rescue the effect of LoF alleles (12). Furthermore, we excluded missense variants from our analyses, although functional characterization can lead to the identification of missense alleles with strong phenotypic effect on human complex phenotypes (27,28).

The main limiting factors of gene KO studies in humans are the sample size and the depth of genetic information available. We have shown that even when the sample size is very large, most gene KOs are identified in single individuals (Supplementary Material, Fig. S3). To be successful, we will need to develop tools to prioritize genes or increase the number of gene KOs. One possibility may be to consider only genes expressed in a tissue that is relevant for the phenotype of interest (e.g. genes expressed in growth plates for height). Another promising solution may be to consider KOs in biological pathways instead of single genes as the testing unit. For instance, a researcher interested in blood lipid genetics could pool together all individuals that carry a gene KO in any of the enzymes or transporters implicated in lipid metabolism. We illustrated this candidate-gene approach by prioritizing OMIM disease-causing genes, genes intolerant to LoF mutations, and genes with relevant mouse KO phenotypes. In particular for the BMI analysis, the enrichment of genes with mouse homologues that disrupt taste or olfaction when inactivated is of interest (Supplementary Material, Fig. S5). Reverse genetic strategies—finding a function to a gene by first disrupting it—have been very successful in model organisms. Despite early challenges, the large-scale identification of LoF variants and characterization of gene KOs promise to also yield interesting insights into human biology.

## Materials and Methods

### Ethics statement

This project was approved by the Ethics Committee of the Montreal Heart Institute (#11-1333, #2013-297, #2013-1438).

### NHLBI Exome Sequence Project

We conducted our initial analysis on the final whole-exome ESP dataset, which is described elsewhere (9). This dataset was generated from high coverage WES (median depth >100×) (9). All study participants in each of the component studies provided written informed consent for the use of their DNA in studies aimed at identifying genetic risk variants for disease and for broad data sharing. Institutional certification was obtained for each sample to allow deposition of phenotype and genotype data in dbGaP and BAM files in the short-read archive. We excluded individuals based on sex mismatch between clinical database and genotype-inferred sex (*N* = 13), high homozygosity (*N* = 1), high genotyping missing rate (>10%) (*N* = 1), if they appear as population outliers in principal component analyses (*N* = 30), low concordance to genome-wide association study data (*N* = 4), or unresolved participant identifiers (*N* = 4). Moreover, we randomly excluded one member of each pair of duplicates (*N* = 16), and of first- and second-degree relatives (*N* = 108). We also removed individuals with chronic obstructive pulmonary disease or asthma, as these conditions could influence anthropometric traits (*N* = 688). Finally, we removed participants from the CARDIA (*N* = 201) and MESA (*N* = 406) studies, as requested by investigators from these studies. We kept individuals aged between 21 and 80 years old, height between 125 and 240 cm, BMI < 75 kg/m^2^, and WHR < 1.5. In total, we analyzed anthropometric traits in 1726 African Americans and 2772 European Americans (Supplementary Material, Table S1).

### Variant quality-control and annotation

We phased variants in the ESP dataset using Beagle 4.0 and the default parameters (19). We define LoF variants as variants that create or remove stop codons (nonsense and stop-loss) that disrupt essential splice sites (two intronic bases at exon-intron boundaries), or that change the reading frame (frameshift indel). We annotated single-base pair variants using in-house custom scripts and build 37.1 of the human genome reference sequence. We annotated frameshift indels using SeattleSeq (hg19, v.9.03, http://snp.gs.washington.edu/SeattleSeqAnnotation138/). We included in our analyses only frameshift indel variants that fall within validated RefSeq genes (release 69). After filtering out variants with a call rate <95% or a Hardy–Weinberg *P* < 1 × 10^−6^, we retained in our analyses 18 137 and 21 935 LoF variants in African- and European-ancestry individuals, respectively (Supplementary Material, Table S1). For comparison, we also annotated ESP variants using Ensembl's Variant Effect Predictor (VEP) module and basic transcripts from GENCODE. We obtained very similar results (Supplementary Material, Fig. S1).

### Replication cohorts with WGS or WES data available

We used low-pass WGS data (mean coverage 5.7×) from 2002 French-Canadian participants recruited by the MHI Biobank. Genotypes were imputed and phased using Beagle 4.0 using the default parameters (19). Individuals were removed due to low or high inbreeding coefficient (*N* = 4). Variants with Hardy–Weinberg *P* < 1 × 10^−8^ were excluded. In total, 1976 MHI Biobank participants with anthropometric traits available were included in the replication analyses (Supplementary Material, Table S1).

### GIANT Consortium ExomeChip datasets

We analyzed Illumina ExomeChip genotype data from 22 studies that are members of the GIANT Consortium (Supplementary Material, Table S1). In total, 103 838, 102 775, and 62 355 individuals were included in the BMI, height and BMI-adjusted WHR analyses, respectively. Individuals were from European- (*N* = 90 927; 19 studies), African- (*N* = 7576; 2 studies), and Hispanic-ancestry (*N* = 5335; 1 study). To increase the number of LoF variants available on the ExomeChip, we broaden our definition of splice-site variants to include variants located two base pairs on either side of exon-intron boundaries. This is the splice-site definition implemented by dbNSFP (29) and used by GIANT across the Consortium's ExomeChip effort. Using the most severe annotation from ENSEMBL's VEP tool, we found that 17.8% (797/4483) of these splice-site variants disrupt a canonical splice-site, 46.7% (2094/4483) are missense variants, and 31.6% (1419/4483) affect a nucleotide around the splice-site (1–3 bases within exon or 3–8 bases within intron). Phasing information was not available for the GIANT exome array data. Because we focused on rare variants, we assumed that when two rare LoF variants were observed in the same gene in the same individual, they were inherited in *trans* to create a compound heterozygous gene KO.

### Statistical analyses

We developed a flexible method to determine if the complete inactivation of genes by LoF variants is associated with human quantitative traits (Fig. 2). For each gene, our method searches for individuals that are either homozygotes or compound heterozygotes for LoF variants; we refer to these individuals as predicted KOs. For X-linked markers that fall outside of the pseudoautosomal regions, we consider predicted gene KOs in men if they carry a single LoF variant. For compound heterozygosity, we use phase information to distinguish LoF variants that segregate on the same haplotype (in *cis*) or on different haplotypes (in *trans*). When phasing information is not available (e.g. GIANT ExomeChip data), we assume that rare LoF variants segregate on different haplotypes. The method then calculates for each gene the phenotypic mean in predicted KO individuals. Finally, it computes statistical significance using phenotype permutations, as follows:

$$\begin{array}{ll} P_{\mathrm{left}}= & \frac{\sum_{i=1}^{n}]\;{]}_{m_{i}\leq m}}{n};P_{\mathrm{right}}=\frac{\sum_{i=1}^{n}]\;{]}_{m_{i}\geq m}}{n}\\ P_{\mathrm{final}}= & 2\times \text{minimum}\;(P_{\mathrm{left}},\;P_{\mathrm{right}}), \end{array}$$

where ]] is the indicator function, *m* is the observed mean phenotype in predicted KO individuals, *m_i_* is the *i*th permuted mean, *n* is the number of permutations, *P*_left_ and *P*_right_ are the left- and right-tail *P*-values, and *P*_final_ is the reported two-tailed *P*-value. Using simulated null phenotypes and the ESP dataset, we showed that the test is well-calibrated (Supplementary Material, Fig. S4). This method assumes that gene inactivation results in the same phenotypic effect (increase or decrease trait value) in all predicted KO individuals for a given gene. The current implementation of our method also currently assumes that tested individuals are unrelated and that the phenotypic distributions are symmetrical. It is compatible with standard genotype file formats (e.g. PLINK, vcf). The scripts to run our method are publicly available at: http://www.mhi-humangenetics.org/en/resources.

### Association of rare predicted gene KOs with anthropometric traits

We analyzed BMI, adult height and BMI-adjusted WHR. We stratified all our analyses by ethnic group, and we only considered rare or low-frequency LoF variants with MAF < 5%. We used 10 000 permutations to assess statistical significance. For genes with an empirical *P* < 2 × 10^−4^ (i.e. permuted means were never higher (or lower) than the observed mean among 10 000 permutations), we re-ran the analysis using 100 000 permutations: only two genes fell in that category (*BRPF1 P*_height_ = 1.8 × 10^−4^; *SPZ1 P*_WHR_ = 2.2 × 10^−4^). For ESP samples, we corrected anthropometric traits for sex, age, ESP phenotype groups, exon capture reagents and the first three principal components, as recommended by the ESP investigators. We then applied inverse normal transformation on the residuals from the previous correction. For the MHI Biobank, and the GIANT studies, each anthropometric trait was corrected for sex, age, age-squared and the first 10 principal components, and we normalized the resulting residuals using inverse normal transformation. Taking into account the direction of the effect, we combined results across studies using a weighted *Z*-score meta-analysis method implemented in the software METAL, where the weight is the sample size of the corresponding study (22). To estimate statistical power of our approach, we modeled the effect of a recessive LoF variant on a normally distributed quantitative trait, as previously described (30). This is a simplistic model as we ignore the presence of additional LoF variants in the same gene, which are considered in our method because they can lead to additional individuals that have a predicted gene KO. We assume that the variant has a MAF = 5%, explains 1% of the genetic variance, and used a sample size of *N* = 4500 (corresponding to ESP), *α* = 2 × 10^−5^ (Bonferroni correction for the number of genes with KOs), and 5000 simulations to perform power calculations. Under this scenario, our gene KO approach would have 95% power to detect the association. Alternatively, testing the association while assuming that the variant has an additive effect would result in only 3% power. Using the same assumptions, we estimated 64 and 1% power for a variant that explains 0.5% of the variance when tested using our gene KO methodology or a simple additive model, respectively.

### Candidate-gene enrichment analyses

We explored whether prioritizing gene KOs into different categories could increase the chance to reveal an association. First, we investigated whether the gene was an OMIM disease-causing gene, as defined elsewhere (26). Next, we considered whether the genes were LoF intolerant by either having a Residual Variation Intolerance Score (RVIS) <15% of the RVIS scores for all genes in the human genome (release 0.3) or a probability of being LoF intolerant (pLI) score >0.9 (20,26). We looked for enrichment by overlapping the QQ-plots of genes belonging to these different categories separately on the QQ-plot containing all genes. We also created subsets of genes based on 30 phenotype categories from the Mouse Genome Informatics (MGI) Database (31). We tested the enrichment using Fisher's exact test.

## Supplementary Material

Supplementary Material is available at *HMG* online.

## Funding

The authors wish to acknowledge the support of the National Heart, Lung, and Blood Institute (NHLBI) and the contributions of the research institutions, study investigators, field staff and study participants in creating this resource for biomedical research. S.L. is funded by a Canadian Institutes of Health Research Banting doctoral scholarship. G.L. is funded by Genome Canada and Génome Québec; the Canada Research Chairs program; and the Montreal Heart Institute Foundation. C.M.L. is supported by Wellcome Trust (grant numbers 086596/Z/08/Z, 086596/Z/08/A); and the Li Ka Shing Foundation. N.S. is funded by National Institutes of Health (grant numbers HL088456, HL111089, HL116747). The Mount Sinai BioMe Biobank Program is supported by the Andrea and Charles Bronfman Philanthropies. GO ESP is supported by NHLBI (RC2 HL-103010 to HeartGO, RC2 HL-102923 to LungGO, RC2 HL-102924 to WHISP). The ESP exome sequencing was performed through NHLBI (RC2 HL-102925 to BroadGO, RC2 HL-102926 to SeattleGO). EGCUT work was supported through the Estonian Genome Center of University of Tartu by the Targeted Financing from the Estonian Ministry of Science and Education (grant number SF0180142s08); the Development Fund of the University of Tartu (grant number SP1GVARENG); the European Regional Development Fund to the Centre of Excellence in Genomics (EXCEGEN) [grant number 3.2.0304.11-0312]; and through FP7 (grant number 313010). EGCUT were further supported by the US National Institute of Health (grant number R01DK075787). A.K.M. was supported by an American Diabetes Association Mentor-Based Postdoctoral Fellowship (#7-12-MN-02). The BioVU dataset used in the analyses described were obtained from Vanderbilt University Medical Centers BioVU which is supported by institutional funding and by the Vanderbilt CTSA grant ULTR000445 from NCATS/NIH. Genome-wide genotyping was funded by NIH grants RC2GM092618 from NIGMS/OD and U01HG004603 from NHGRI/NIGMS. Funding to pay the Open Access publication charges for this article was provided by a block grant from Research Councils UK to the University of Cambridge.

## Supplementary Material

Supplementary Data
