# Supplementary material for: Testing the role of predicted gene knockouts in human anthropometric trait variation
Source: Hum Mol Genet. 2016 Feb 21;25(10):2082–92. doi: 10.1093/hmg/ddw055 (PMC5062577; doi:10.1093/hmg/ddw055)
Supplement: Supplementary Data [file supp_25_10_2082__index.html]

Testing the Role of Predicted Gene Knockouts in Human Anthropometric Trait Variation — Testing the role of predicted gene knockouts in human anthropometric trait variation — Testing the Role of Predicted Gene Knockouts in Human Anthropometric Trait Variation — Supplementary Data 

# Testing the role of predicted gene knockouts in human anthropometric trait variation

## Supplementary Data

Supplementary Data

- Supplementary Data - Docx file
- Supplementary Table 3 - xlsx file
- Supplementary Table 4 - xlsx file
