# Supplementary material for: Testing the role of predicted gene knockouts in human anthropometric trait variation
Source: Hum Mol Genet. 2016 Feb 21;25(10):2082–92. doi: 10.1093/hmg/ddw055 (PMC5062577; doi:10.1093/hmg/ddw055)
Supplement: Supplementary Data [file supp_ddw055_ddw055supp.docx]

**SUPPLEMENTAL DATA**

**Table S1.** Descriptive statistics for the different studies analyzed in this project. For age, height, body mass index (BMI) and waist-hip ratio adjusted for BMI (WHR), we provide the mean trait value by sex. For the GIANT ExomeChip studies, we provide the maximum number of loss-of-function (LoF) variants available based on the array content. Rare LoF variants have a minor allele frequency <5% (for the ExomeChip datasets, we used allele frequencies from the MHI Biobank to fill in this table). AA, African Americans; EA, Europeans or individuals of European-ancestry. NA, not available.

| **Study (ethnicity)** | **Sample size**  **(men / women)** | **Age**  **(men / women)** | **Height (cm)**  **(men / women)** | **BMI (kg/m^2^)**  **(men / women)** | **WHR**  **(men / women)** | **Number of loss-of-function (LoF) variants**  **(all / rare)** | | | |
| --- | --- | --- | --- | --- | --- | --- | --- | --- | --- |
|  |  |  |  |  |  | **Nonsense** | **Stop-loss** | **Splice site** | **Frameshift indel** |
| ***NHLBI Exome Sequence Project (ESP)*** | | | | | | | | | |
| ESP (AA) | 372 / 1354 | 54.6 / 58.2 | 176.3 / 162.7 | 28,9 / 35.2 | 0.95 / 0.84 | 5787 / 5611 | 288 / 249 | 2705 / 2584 | 9357 / 9002 |
| ESP (EA) | 994 / 1778 | 50.5 / 58.9 | 176.6 / 161.7 | 28.4 / 28.2 | 0.97 / 0.84 | 7773 / 7623 | 274 / 242 | 3500 / 3398 | 10388 / 10088 |
| ***MHI Biobank whole-genome sequencing*** | | | | | | | | | |
| MHI (EA) | 1436 / 531 | 65.1 / 69.7 | 172.7 / 157.4 | 29.4 / 28.9 | 0.99 / 0.88 | 3399 / 3361 | 211 / 187 | 1761 / 1710 | 3397 / 3174 |
| ***GIANT ExomeChip studies*** | | | | | | | | | |
| ARIC (AA) | 1271 / 2086 | 53.9 / 53.5 | 176.1 / 163.1 | 27.7 / 31.0 | 0.94 / 0.91 | 5658 / 2136 | 244 / 93 | 10667 / 4483 | NA |
| ARIC (EA) | 5102 / 5771 | 54.7 / 54.0 | 176.2 / 162.0 | 27.4 / 26.6 | 0.97 / 0.89 |  |  |  | NA |
| ASCOT-SC (EA) | 1833 / 629 | 62.5 / 64.4 | 176 / 160 | 28.6 / 28.9 | NA |  |  |  | NA |
| ASCOT-UK (EA) | 2656 / 587 | 65/63 | 174 / 161 | 29 / 28 | NA |  |  |  | NA |
| BioME (AA) | 1530 / 2682 | 52.8 / 54.1 | 177.9 / 163.9 | 28.5 / 31.6 | NA |  |  |  | NA |
| BioME (EA) | 1475 / 1135 | 66.3 / 66.0 | 177.2 / 162.5 | 27.7 / 26.2 | NA |  |  |  | NA |
| BioME (HA) | 1980 / 3355 | 54.3 / 56.2 | 172.1 / 159.4 | 28.9 / 29.8 | NA |  |  |  | NA |
| BIOVU (EA) | 9535 / 11391 | 58.1 / 55.4 | 178.5 / 163.3 | 28.3 / 27.9 | NA |  |  |  | NA |
| BRIGHT (EA) | 308 / 480 | 57.4 / 58.6 | 173.4 / 160.8 | 27.7 / 27.4 | 0.94 / 0.83 |  |  |  | NA |
| EFSOCH (EA) | 726 / 794 | 32.9 / 30.4 | 177.7 / 165.0 | 26.6 / 25.0 | 0.89 / 0.81 |  |  |  | NA |
| EGCUT (EA) | 2041 / 2384 | 48.6 / 49.2 | 178.2 / 164 | 28.5 / 28.7 | 0.95 / 0.82 |  |  |  | NA |
| Endo (EA) | 0 / 533 | NA / 37.7 | NA / NA | 0 / 24.4 | NA / NA |  |  |  | NA |
| EXTEND (EA) | 610 / 971 | 58.4 / 55.33 | 175.9 / 163.2 | 28.1 / 26.8 | 0.94 / 0.81 |  |  |  | NA |
| InterAct – Cases (EA) | 1289 / 1352 | 55.0 / 55.8 | 173.5 / 160.3 | 29.3 / 30.3 | 0.98 / 0.85 |  |  |  | NA |
| InterAct – Subcohort (EA) | 1644 / 3085 | 51.5 / 51.7 | 174.0 / 161.4 | 26.4 / 25.5 | 0.93 / 0.80 |  |  |  | NA |
| MHI (EA) | 5629 / 3956 | 64.4 / 62.8 | 172.4 / 159.2 | 28.9 / 28.0 | 0.98 / 0.87 |  |  |  | NA |
| PICOS (EA) | 0 / 582 | NA / 32.1 | NA / 165.0 | NA / 28.0 | NA / 0.80 |  |  |  | NA |
| PIVUS – men (EA) | 487 / 0 | 70.1 / NA | 175.9 / NA | 27.0 / NA | 0.94 / NA |  |  |  | NA |
| PIVUS – women (EA) | 0 / 474 | NA / 70.3 | NA / 162.3 | NA / 27.1 | NA / 0.86 |  |  |  | NA |
| ULSAM | 1102 / 0 | 71.0 / NA | 174.9 / NA | 26.2 / NA | 0.94 / NA |  |  |  | NA |
| RISC (EA) | 156 / 157 | 44.7 / 45.8 | 178.5 / 164.9 | 26.0 / 25.2 | 0.93 / 0.82 |  |  |  | NA |
| WHI (EA) | 0 / 22125 | NA / 66.2 | NA / 161.5 | NA / 28.3 | NA / 0.82 |  |  |  | NA |

**Table S2.** Number and frequency of predicted gene knockouts (KOs) in 1,785 African Americans and 2,896 European Americans from the NHLBI Exome Sequence Project (ESP). In comparison with **Table 1**, we consider in this loss-of-function (LoF) variant analysis autosomal nonsense, stop-loss and splice site variants, but excluded frameshift insertion-deletions (indels). Rare LoF variants have a minor allele frequency <5%. In the absence of phasing information, we assume that rare LoF are inherited in *trans*. As we can see, considering phased genotype information significantly impacts the number of gene KOs that we can detect due to compound heterozygosity.

|  |  |  |  | **NOT PHASED** | | **PHASED** | |
| --- | --- | --- | --- | --- | --- | --- | --- |
|  |  | ***Variants***  ***/individuals*** | ***Variants***  ***/gene*** | ***KO Events***  ***/Individuals*** | ***Number of KO Genes*** | ***KO Events***  ***/Individuals*** | ***Number of KO Genes*** |
| African-Americans | All LoF (N=8,802) | 112.4 | 0.45 | 12.9 | 497 | 12.3 | 474 |
|  | Homozygotes |  |  | 11.6 | 447 | 11.6 | 447 |
|  | Compound heterozygotes |  |  | 1.32 | 156 | 0.71 | 112 |
|  | Rare LoF (N=8,505) | 25.7 | 0.43 | 0.41 | 275 | 0.29 | 255 |
|  | Homozygotes |  |  | 0.24 | 230 | 0.24 | 230 |
|  | Compound heterozygotes |  |  | 0.17 | 70 | 0.05 | 47 |
| European-Americans | All LoF (N=11,687) | 92.1 | 0.60 | 12.0 | 342 | 11.7 | 328 |
|  | Homozygotes |  |  | 11.1 | 317 | 11.1 | 317 |
|  | Compound heterozygotes |  |  | 0.89 | 103 | 0.59 | 74 |
|  | Rare LoF (N=11,437) | 15.5 | 0.58 | 0.15 | 171 | 0.14 | 158 |
|  | Homozygotes |  |  | 0.13 | 151 | 0.13 | 151 |
|  | Compound heterozygotes |  |  | 0.03 | 37 | 0.01 | 20 |

**Table S3.** Gene KO association results with anthropometric trait in ESP WES and MHI Biobank WGS datasets.

This table is available in a separate Excel file.

**Table S4.** Gene KO association results with anthropometric trait using exome array data from studies involved in the GIANT Consortium.

This table is available in a separate Excel file.

**Figure S1.** Comparison of association *P*-values using different variant annotations in the ESP dataset for (**A**) BMI, (**B**) height, and (**C**) WHR. On the *x*-axis, the analysis was performed on variants that fell within validated Refseq genes (release 69) annotated as a frameshift indel, as creating or removing a stop codon (nonsense or stop-loss), or disturbing an essential splice-site. These results are equivalent to the analyses reported in **Figure 3** (**A-C**) of the main manuscript. On the y-axis, variants were chosen to fall within GENCODE basic transcripts (GENCODE version 19), and annotated as having a high impact using the ENSEMBL variant effect predictor tool. Only those predicted as high confidence by the Loss-Of-Function Transcript Effect Estimator (LOFTEE) plugin were kept. In all cases, we observed high correlation between *P*-values generated using both annotations (BMI *r^2^*=0.929, *P*<2x10^-16^; Height *r^2^*=0.930, *P*<2x10^-16^; WHR *r^2^*=0.931, *P*<2x10^-16^). No genes reached statistical significance using the GENCODE annotation.

**
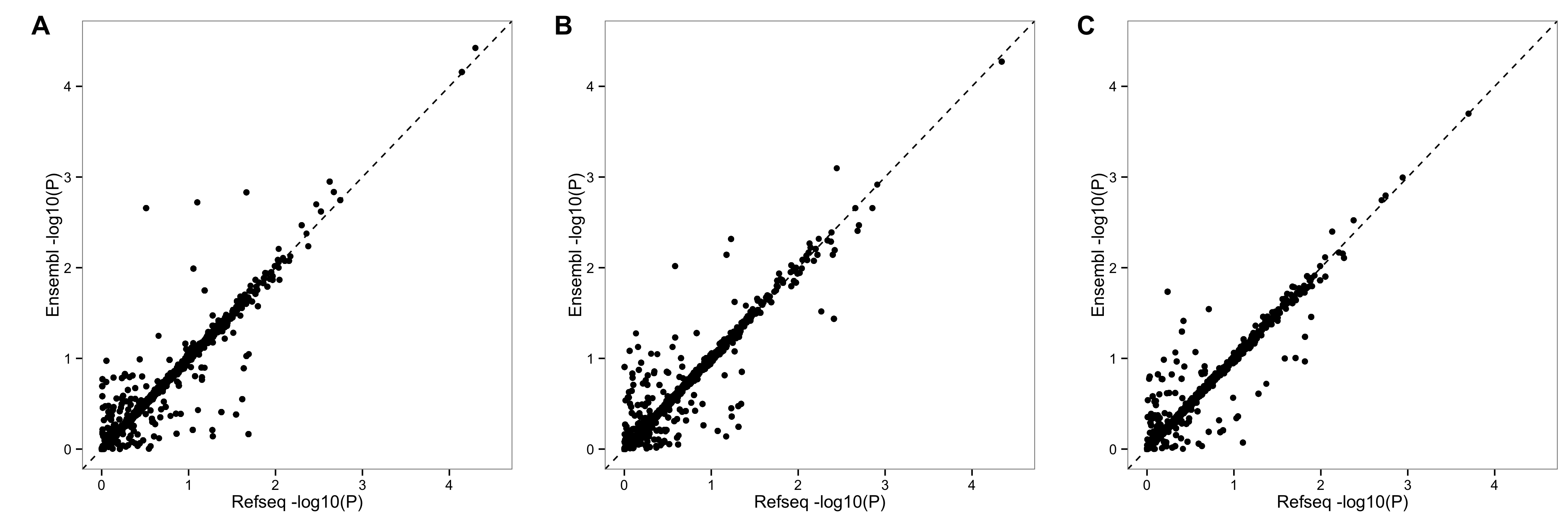
**

**Figure S2.** Number of predicted knockout (KO) genes per NHLBI Exome Sequence Project participant. We present distributions in African Americans (**A** and **B**) and European Americans (**C** and **D**) separately. For these analyses, we did not include frameshift indels. We include common and rare loss-of-function (LoF: nonsense, stop-loss, splice site) variants in **A** and **C**, whereas only rare LoF variants (minor allele frequency <5%) are included in **B** and **D**. Homo.: gene KO due to homozygosity; Comp. het.: gene KO due to compound heterozygosity; Both: genes with homozygous and compound heterozygous LoF variants.

**Figure S3.** Frequency of KO events in the ESP and GIANT datasets. Frequency of KOs in individuals of African ancestry are shown for the ESP **(A)** and GIANT **(B)** datasets. Frequency of KOs in individuals of European ancestry are shown for the ESP **(C)** and GIANT **(D)** datasets. As we can see in Europe-ancestry individuals from the GIANT studies, increasing the sample size mostly increases singletons, that is genes that are KO in a single individual. N: number of individuals.

**Figure S4.** Calibration of our statistical method using the NHLBI Exome Sequence Project (ESP) dataset. We present quantile-quantile (QQ) plots of associations between predicted KO genes in African Americans (A) and European Americans (B), and 100 randomly generated quantitative phenotypes (mean=0, standard deviation (SD)=1). For each simulation, we ranked P-values and calculated the mean of each rank across simulations. The dots represent this mean, whereas the dotted lines are mean ± 1 SD. We also calculated the mean GC ± SD. As expected, the genomic inflation factor (λ_GC_) is ~1.

**
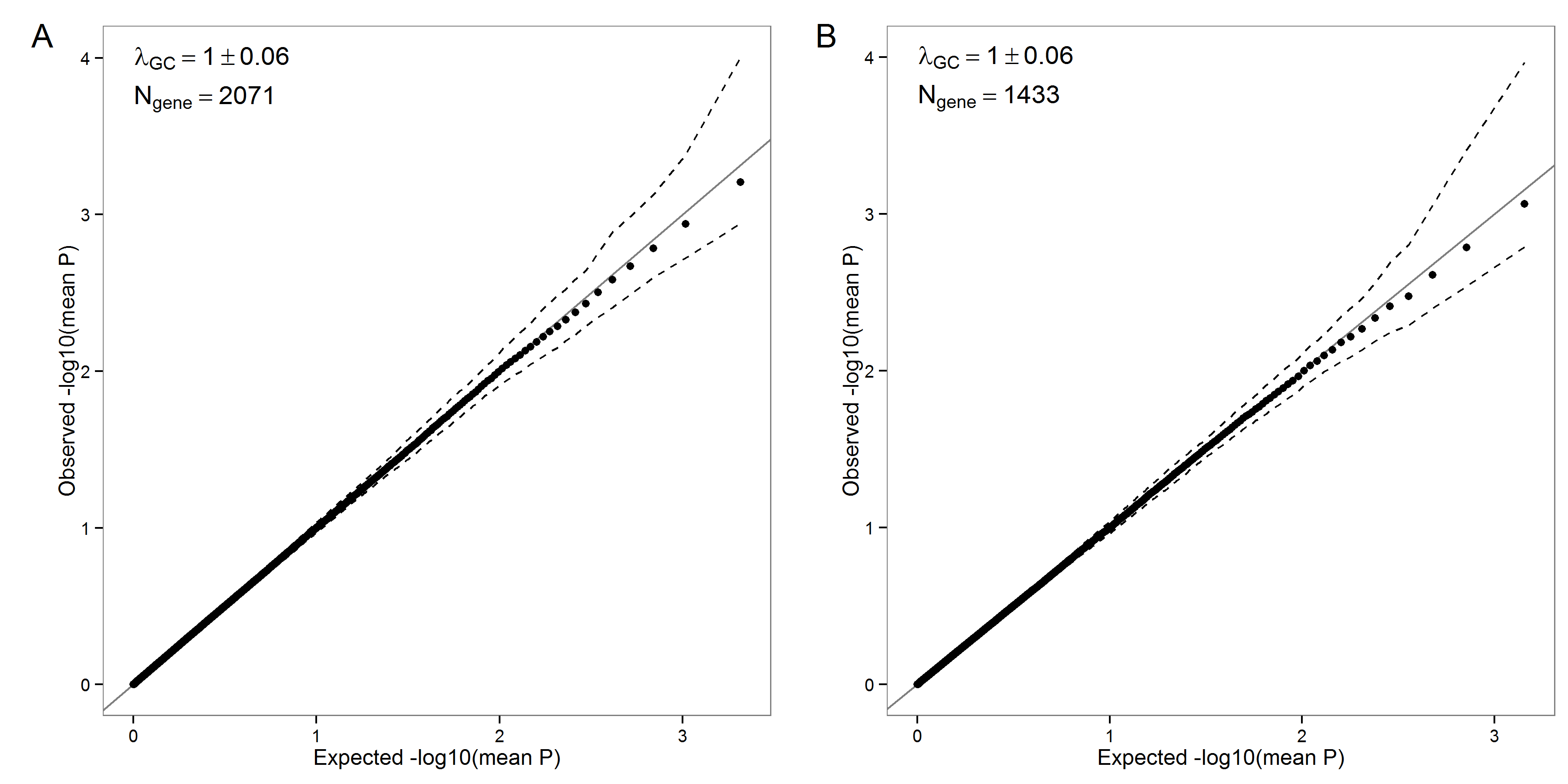
**

**Figure S5.** Quantile-quantile (QQ) plots of association results between predicted gene knockouts (KOs) and anthropometric traits restricted to 30 phenotype categories from the Mouse Genome Informatics (MGI) database in the (**A-C**) NHLBI Exome Sequence Project (ESP) and (**D-F**) GIANT ExomeChip datasets. We analyzed three anthropometric traits: (**A**) BMI (N_participants_=4,475), (**B**) height (N_participants_=4,423), and (**C**) WHR (N_participants_=2,973). We analyzed the same traits in the GIANT dataset: (**D**) BMI (N_participants_=103,838), (**E**) height (N_participants_=102,775), and (**F**) WHR (N_participants_=62,355). Results are not corrected for the genomic inflation factor. The dash lines correspond to the 95% confidence interval. λ_GC_, genomic inflation factor; N_gene_, number of genes with at least one participant that carries two LoF alleles.

**Figure S6.** Comparison of association *P*-values using different variant annotations in the ESP dataset for (**A**) BMI, (**B**) height, and (**C**) WHR. On the *x*-axis, the analysis includes all KO events (compound heterozygotes and homozygotes). On the *y*-axis, compound heterozygotes were excluded. These results are equivalent to the analyses reported in **Figure 3** (**A-C**) of the main text. In all cases, we observed high correlation between *P*-values generated using both annotations (BMI *r^2^*= 0.944, *P*<2x10^-16^; Height *r^2^*=0.943, *P*<2x10^-16^; WHR *r^2^*=0.942, *P*<2x10^-16^).

**Further acknowledgements**

**HeartGO:**

**Atherosclerosis Risk in Communities (ARIC)**: NHLBI (HHSN268201100005C, HHSN268201100006C, HHSN268201100007C, HHSN268201100008C, HHSN268201100009C, HHSN268201100010C, HHSN268201100011C, and HHSN268201100012C); **Cardiovascular Health Study (CHS)**: NHLBI contracts HHSN268201200036C, HHSN268200800007C, N01HC55222, N01HC85079, N01HC85080, N01HC85081, N01HC85082, N01HC85083, N01HC85086; and NHLBI grants U01HL080295, R01HL087652, R01HL105756, R01HL103612, R01DK089256, and R01HL120393 with additional contribution from the National Institute of Neurological Disorders and Stroke (NINDS). Additional support was provided through R01AG023629 from the National Institute on Aging (NIA); **Coronary Artery Risk Development in Young Adults (CARDIA)**: NHLBI (N01-HC95095 & N01-HC48047, N01-HC48048, N01-HC48049, and N01-HC48050); **Framingham Heart Study (FHS)**: NHLBI (N01-HC-25195 and grant R01 NS17950) with additional support from NIA (AG08122 and AG033193); **Jackson Heart Study (JHS)**: NHLBI and the National Institute on Minority Health and Health Disparities (N01 HC-95170, N01 HC-95171 and N01 HC-95172); **Multi-Ethnic Study of Atherosclerosis (MESA**)**:** NHLBI (N01-HC-95159 through N01-HC-95169 and RR-024156).

**Lung GO:**

**Cystic Fibrosis (CF):** Cystic Fibrosis Foundation (GIBSON07K0, KNOWLE00A0, OBSERV04K0, RDP R026), the NHLBI (R01 HL-068890, R02 HL-095396), NIH National Center for Research Resources (UL1 RR-025014), and the National Human Genome Research Institute (NHGRI) (5R00 HG-004316). **Chronic Obstructive Pulmonary Disease (COPDGene):** NHLBI (U01 HL-089897, U01 HL-089856), and the COPD Foundation through contributions made to an Industry Advisory Board comprised of AstraZeneca, Boehringer Ingelheim, Novartis, Pfizer, and Sunovian. The COPDGene clinical centers and investigators are available at www.copdgene.org. **Acute Lung Injury (ALI)**: NHLBI (RC2 HL-101779). **Lung Health Study (LHS)**: NHLBI (RC2 HL-066583), the NHGRI (HG-004738), and the NHLBI Division of Lung Diseases (HR-46002). **Pulmonary Arterial Hypertension (PAH):** NIH (P50 HL-084946, K23 AR-52742), and the NHLBI (F32 HL-083714). **Asthma:** NHLBI (RC2 HL-101651), and the NIH (HL-077916, HL-69197, HL-76285, M01 RR-07122).

**SWISS and ISGS:**

Siblings with Ischemic Stroke Study (SWISS): National Institute of Neurological Disorders and Stroke (NINDS) (R01 NS039987); Ischemic Stroke Genetics Study (ISGS): NINDS (R01 NS042733)

**WHISP:**

**Women’s Health Initiative (WHI):** The WHI Sequencing Project is funded by the National Heart, Lung, and Blood Institute (HL-102924) as well as the National Institutes of Health (NIH), U.S. Department of Health and Human Services through contracts HHSN268201100046C, HHSN268201100001C, HHSN268201100002C, HHSN268201100003C, HHSN268201100004C, and HHSN271201100004C. The authors thank the WHI investigators and staff for their dedication, and the study participants for making the program possible. A full listing of WHI investigators can be found at: [*www.whi.org/researchers/Documents%20%20Write%20a%20Paper/WHI%20Investigator%20Short%20List.pdf*](http://www.whi.org/researchers/Documents%20%20Write%20a%20Paper/WHI%20Investigator%20Short%20List.pdf)

**NHLBI GO Exome Sequencing Project**

**BroadGO**

Stacey B. Gabriel (Broad Institute)^4, 5, 11, 16, 17^, David M. Altshuler (Broad Institute, Harvard Medical School, Massachusetts General Hospital)^1, 5, 7, 17^, Gonçalo R. Abecasis (University of Michigan)^3, 5, 9, 13, 15, 17^, Hooman Allayee (University of Southern California)^5^, Sharon Cresci (Washington University School of Medicine)^5^, Mark J. Daly (Broad Institute, Massachusetts General Hospital), Paul I. W. de Bakker (Broad Institute, Harvard Medical School, University Medical Center Utrecht)^3, 15^, Mark A. DePristo (Broad Institute)^4, 13, 15, 16^, Ron Do (Broad Institute)^5, 9, 13, 15^, Peter Donnelly (University of Oxford)^5^, Deborah N. Farlow (Broad Institute)^3, 4, 5, 12, 14, 16, 17^, Tim Fennell (Broad Institute), Kiran Garimella (University of Oxford)^4, 16^, Stanley L. Hazen (Cleveland Clinic)^5^, Youna Hu (University of Michigan)^3, 9, 15^, Daniel M. Jordan (Harvard Medical School, Harvard University)^13^, Goo Jun (University of Michigan)^13^, Sekar Kathiresan (Broad Institute, Harvard Medical School, Massachusetts General Hospital)^5, 8, 9, 12, 14, 15, 17, 20^, Hyun Min Kang (University of Michigan)^9, 13, 16^, Adam Kiezun (Broad Institute)^5, 13, 15^, Guillaume Lettre (Broad Institute, Montreal Heart Institute, Université de Montréal)^1, 2, 13, 15^, Bingshan Li (University of Michigan)^3^, Mingyao Li (University of Pennsylvania)^5^, Christopher H. Newton-Cheh (Broad Institute, Massachusetts General Hospital, Harvard Medical School)^3, 8, 15^, Sandosh Padmanabhan (University of Glasgow School of Medicine)^3, 12, 15^, Gina Peloso (Broad Institute, Harvard Medical School, Massachusetts General Hospital)^5^, Sara Pulit (Broad Institute)^3, 15^, Daniel J. Rader (University of Pennsylvania)^5^, David Reich (Broad Institute, Harvard Medical School)^15^, Muredach P. Reilly (University of Pennsylvania)^5^, Manuel A. Rivas (Broad Institute, Massachusetts General Hospital)^5^, Steve Schwartz (Fred Hutchinson Cancer Research Center)^5, 12^, Laura Scott (University of Michigan)^1^, David S. Siscovick (University of Washington)^5, 1, 25^, John A. Spertus (University of Missouri Kansas City)^5^, Nathan O. Stitziel (Brigham and Women's Hospital)^5, 15^, Nina Stoletzki (Brigham and Women's Hospital, Broad Institute, Harvard Medical School)^13^, Shamil R. Sunyaev (Brigham and Women's Hospital, Broad Institute, Harvard Medical School)^1, 3, 5, 13, 15^, Benjamin F. Voight (Broad Institute, Massachusetts General Hospital), Cristen J. Willer (University of Michigan)^1, 9, 13, 15^

**HeartGO**

Stephen S. Rich (University of Virginia)^2, 4, 7, 8, 9, 11,14, 15, 17, 18, 31^, Ermeg Akylbekova (Jackson State University, University of Mississippi Medical Center)^29^, Larry D. Atwood* (Boston University)^1, 11, 28^, Christie M. Ballantyne (Baylor College of Medicine, Methodist DeBakey Heart Center)^9, 22^, Maja Barbalic (University of Texas Health Science Center Houston)^9, 14, 15, 17, 22^, R. Graham Barr (Columbia University Medical Center)^10, 31^, Emelia J. Benjamin (Boston University)^14, 20, 28^, Joshua Bis (University of Washington)^15, 23^, Eric Boerwinkle (University of Texas Health Science Center Houston)^3, 5, 9, 13, 15, 17, 22^, Donald W. Bowden (Wake Forest University)^1, 31^, Jennifer Brody (University of Washington)^3, 5, 15, 23^, Matthew Budoff (Harbor-UCLA Medical Center)^31^, Greg Burke (Wake Forest University)^5, 31^, Sarah Buxbaum (Jackson State University)^3, 13, 15, 29^, Jeff Carr (Wake Forest University)^25, 29, 31^, Donna T. Chen (University of Virginia)^6, 11^, Ida Y. Chen (Cedars-Sinai Medical Center)^1, 31^, Wei-Min Chen (University of Virginia)^13, 15, 18^, Pat Concannon (University of Virginia)^11^, Jacy Crosby (University of Texas Health Science Center Houston)^22^, L. Adrienne Cupples (Boston University)^1, 3, 5, 9, 13, 15, 18, 28^, Ralph D'Agostino (Boston University)^28^, Anita L. DeStefano (Boston University)^13, 18^, ^28^, Albert Dreisbach (University of Mississippi Medical Center)^3, 29^, Josée Dupuis (Boston University)^1, 28^, J. Peter Durda (University of Vermont)^15, 23^, Jaclyn Ellis (University of North Carolina Chapel Hill)^1^, Aaron R. Folsom (University of Minnesota)^5^, ^22^, Myriam Fornage (University of Texas Health Science Center Houston)^3, 18, 25^, Caroline S. Fox (National Heart, Lung, and Blood Institute)^1, 28^, Ervin Fox (University of Mississippi Medical Center)^3, 9, 29^, Vincent Funari (Cedars-Sinai Medical Center)^1, 11, 31^, Santhi K. Ganesh (University of Michigan)^2, 22^, Julius Gardin (Hackensack University Medical Center)^25^, David Goff (Wake Forest University)^25^, Ora Gordon (Cedars-Sinai Medical Center)^11, 31^, Wayne Grody (University of California Los Angeles)^11, 31^, Myron Gross (University of Minnesota)^1, 5, 14, 25^, Xiuqing Guo (Cedars-Sinai Medical Center)^3, 15, 31^, Ira M. Hall (University of Virginia), Nancy L. Heard-Costa (Boston University)^1, 11, 28^, Susan R. Heckbert (University of Washington)^10, 14, 20, 23^, Nicholas Heintz (University of Vermont), David M. Herrington (Wake Forest University)^5, 31^, DeMarc Hickson (Jackson State University, University of Mississippi Medical Center)^29^, Jie Huang (National Heart, Lung, and Blood Institute)^5, 28^, Shih-Jen Hwang (Boston University, National Heart, Lung, and Blood Institute)^3, 28^, David R. Jacobs (University of Minnesota)^25^, Nancy S. Jenny (University of Vermont)^1, 2, 23^, Andrew D. Johnson (National Heart, Lung, and Blood Institute)^2, 5, 11, 28^, Craig W. Johnson (University of Washington)^15, 31^, Steven Kawut (University of Pennsylvania)^10,31^, Richard Kronmal (University of Washington)^31^, Raluca Kurz (Cedars-Sinai Medical Center)^11, 31^, Ethan M. Lange (University of North Carolina Chapel Hill)^3, 5, 9, 13, 34^, Leslie A. Lange (University of North Carolina Chapel Hill)^1, 2, 3, 5, 9, 12, 13, 15, 17, 18, 20, 25, 34^, Martin G. Larson (Boston University)^3, 15, 28^, Mark Lawson (University of Virginia), Cora E. Lewis (University of Alabama at Birmingham)^25,34^, Daniel Levy (National Heart, Lung, and Blood Institute)^3, 15, 17, 28^, Dalin Li (Cedars-Sinai Medical Center)^11, 15, 31^, Honghuang Lin (Boston University)^20, 28^, Chunyu Liu (National Heart, Lung, and Blood Institute)^3, 28^, Jiankang Liu (University of Mississippi Medical Center)^1, 29^, Kiang Liu (Northwestern University)^25^, Xiaoming Liu (University of Texas Health Science Center Houston)^15, 22^, Yongmei Liu (Wake Forest University)^2, 5, 31^, William T. Longstreth (University of Washington)^18, 23^, Cay Loria (National Heart, Lung, and Blood Institute)^25^, Thomas Lumley (University of Auckland)^9, 23^, Kathryn Lunetta (Boston University)^28^, Aaron J. Mackey (University of Virginia)^16, 18^, Rachel Mackey (University of Pittsburgh)^1, 23, 31^, Ani Manichaikul (University of Virginia)^8, 15, 18, 31^, Taylor Maxwell (University of Texas Health Science Center Houston)^22^, Barbara McKnight (University of Washington)^15, 23^, James B. Meigs (Brigham and Women's Hospital, Harvard Medical School, Massachusetts General Hospital)^1, 28^, Alanna C. Morrison (University of Texas Health Science Center Houston)^3, 15, 17^, Solomon K. Musani (University of Mississippi Medical Center)^3, 29^, Josyf C. Mychaleckyj (University of Virginia)^13, 15, 31^, Jennifer A. Nettleton (University of Texas Health Science Center Houston)^9, 22^, Kari North (University of North Carolina Chapel Hill)^1, 3, 9, 10, 13, 15, 17, 34^, Christopher J. O'Donnell (Massachusetts General Hospital, National Heart, Lung, and Blood Institute)^2, 5, 9, 11, 12, 14, 15, 17, 20, 28^, Daniel O'Leary (Tufts University School of Medicine)^25, 31^, Frank S. Ong (Cedars-Sinai Medical Center)^3, 11, 31^, Walter Palmas (Columbia University)^3, 15, 31^, James S. Pankow (University of Minnesota)^1, 22^, Nathan D. Pankratz (Indiana University School of Medicine)^15, 25^, Shom Paul (University of Virginia), Marco Perez (Stanford University School of Medicine), Sharina D. Person (University of Alabama at Birmingham, University of Alabama at Tuscaloosa)^25^, Joseph Polak (Tufts University School of Medicine)^31^, Wendy S. Post (Johns Hopkins University)^3, 9, 11, 14, 20, 31^, Bruce M. Psaty (Group Health Research Institute, University of Washington)^3, 5, 9, 11, 14, 15, 23^, Aaron R. Quinlan (University of Virginia)^18, 19^, Leslie J. Raffel (Cedars-Sinai Medical Center)^6, 11, 31^, Vasan S. Ramachandran (Boston University)^3, 28^, Alexander P. Reiner (Fred Hutchinson Cancer Research Center, University of Washington)^1, 2, 3, 5, 9, 11, 12, 13, 14, 15, 20, 25, 34^, Kenneth Rice (University of Washington)^15, 23^, Jerome I. Rotter (Cedars-Sinai Medical Center)^1, 3, 6, 8, 11, 15, 31^, Jill P. Sanders (University of Vermont)^23^, Pamela Schreiner (University of Minnesota)^25^, Sudha Seshadri (Boston University)^18, 28^, Steve Shea (Brigham and Women's Hospital, Harvard University)^28^, Stephen Sidney (Kaiser Permanente Division of Research, Oakland, CA)^25^, Kevin Silverstein (University of Minnesota)^25^, David S. Siscovick (University of Washington)^5, 1, 25^, Nicholas L. Smith (University of Washington)^2, 15, 20, 23^, Nona Sotoodehnia (University of Washington)^3, 15, 23^, Asoke Srinivasan (Tougaloo College)^29^, Herman A. Taylor (Jackson State University, Tougaloo College, University of Mississippi Medical Center)^5,^ ^29^, Kent Taylor (Cedars-Sinai Medical Center)^31^, Fridtjof Thomas (University of Texas Health Science Center Houston)^3, 22^, Russell P. Tracy (University of Vermont)^5, 9, 11, 12, 14, 15, 17, 20, 23^, Michael Y. Tsai (University of Minnesota)^9, 31^, Kelly A. Volcik (University of Texas Health Science Center Houston)^22^, Chrstina L Wassel (University of California San Diego)^9, 15, 31^, Karol Watson (University of California Los Angeles)^31^, Gina Wei (National Heart, Lung, and Blood Institute)^25^, Wendy White (Tougaloo College)^29^, Kerri L. Wiggins (University of Vermont)^23^, Jemma B. Wilk (Boston University)^10, 28^, O. Dale Williams (Florida International University)^25^, Gregory Wilson (Jackson State University)^29^, James G. Wilson (University of Mississippi Medical Center)^1, 2, 5, 8, 9, 11, 12, 14, 17, 20, 29^, Phillip Wolf (Boston University)^28^, Neil A. Zakai (University of Vermont)^2, 23^

**ISGS and SWISS**

John Hardy (Reta Lila Weston Research Laboratories, Institute of Neurology, University College London)^18^, James F. Meschia (Mayo Clinic)^18^, Michael Nalls (National Institute on Aging)^2, 18^, Stephen S. Rich (University of Virginia)^2, 4, 7, 8, 9, 11, 14, 15, 17, 18, 31^, Andrew Singleton (National Institute on Aging)^18^, Brad Worrall (University of Virginia)^18^

**LungGO**

Michael J. Bamshad (Seattle Children's Hospital, University of Washington)^4, 6, 7, 8, 10, 11, 13, 15, 17, 27^, Kathleen C. Barnes (Johns Hopkins University)^2, 10, 12, 14, 15, 17, 20, 24, 30, 32^, Ibrahim Abdulhamid (Children’s Hospital of Michigan)^27^, Frank Accurso (University of Colorado)^27^, Ran Anbar (Upstate Medical University)^27^, Terri Beaty (Johns Hopkins University)^24, 30^, Abigail Bigham (University of Washington)^13, 15, 27^, Phillip Black (Children’s Mercy Hospital)^27^, Eugene Bleecker (Wake Forest University)^33^, Kati Buckingham (University of Washington)^27^, Anne Marie Cairns (Maine Medical Center)^27^, Wei-Min Chen (University of Virginia)^13, 15, 18^, Daniel Caplan (Emory University)^27^, Barbara Chatfield (University of Utah)^27^, Aaron Chidekel (A.I. Dupont Institute Medical Center)^27^, Michael Cho (Brigham and Women's Hospital, Harvard Medical School)^13, 15, 24^, David C. Christiani (Massachusetts General Hospital)^21^, James D. Crapo (National Jewish Health)^24, 30^, Julia Crouch (Seattle Children's Hospital)6, Denise Daley (University of British Columbia)^30^, Anthony Dang (University of North Carolina Chapel Hill)^26^, Hong Dang (University of North Carolina Chapel Hill)^26^, Alicia De Paula (Ochsner Health System)^27^, Joan DeCelie-Germana (Schneider Children’s Hospital)^27^, Allen Dozor (New York Medical College, Westchester Medical Center)^27,^ Mitch Drumm (University of North Carolina Chapel Hill)^26^, Maynard Dyson (Cook Children’s Med. Center)^27^, Julia Emerson (Seattle Children's Hospital, University of Washington)^27^, Mary J. Emond (University of Washington)^10, 13, 15, 17, 27^, Thomas Ferkol (St. Louis Children's Hospital, Washington University School of Medicine)^27^, Robert Fink (Children’s Medical Center of Dayton)^27^, Cassandra Foster (Johns Hopkins University)^30^, Deborah Froh (University of Virginia)^27^, Li Gao (Johns Hopkins University)^24, 30, 32^, William Gershan (Children’s Hospital of Wisconsin)^27^, Ronald L. Gibson (Seattle Children's Hospital, University of Washington)^10, 27^, Elizabeth Godwin (University of North Carolina Chapel Hill)^26^, Magdalen Gondor (All Children’s Hospital Cystic Fibrosis Center)^27^, Hector Gutierrez (University of Alabama at Birmingham)^27^, Nadia N. Hansel (Johns Hopkins University, Johns Hopkins University School of Public Health)^10, 15, 30^, Paul M. Hassoun (Johns Hopkins University)^10, 14, 32^, Peter Hiatt (Texas Children's Hospital)^27^, John E. Hokanson (University of Colorado)^24^, Michelle Howenstine (Indiana University, Riley Hospital for Children)^27^, Laura K. Hummer (Johns Hopkins University)^32^, Seema M. Jamal (University of Washington)^11^, Jamshed Kanga (University of Kentucky)^27^, Yoonhee Kim (National Human Genome Research Institute)^24, 32^, Michael R. Knowles (University of North Carolina Chapel Hill)^10, 26^, Michael Konstan (Rainbow Babies & Children’s Hospital)^27^, Thomas Lahiri (Vermont Children’s Hospital at Fletcher Allen Health Care)^27^, Nan Laird (Harvard School of Public Health)^24^, Christoph Lange (Harvard School of Public Health)^24^, Lin Lin (Harvard Medical School)^21^, Xihong Lin (Harvard School of Public Health)^21^, Tin L. Louie (University of Washington)^13, 15, 27^, David Lynch (National Jewish Health)^24^, Barry Make (National Jewish Health)^24^, Thomas R. Martin (University of Washington, VA Puget Sound Medical Center)^10, 21^, Steve C. Mathai (Johns Hopkins University)^32^, Rasika A. Mathias (Johns Hopkins University)^10, 13, 15, 30, 32^, John McNamara (Children’s Hospitals and Clinics of Minnesota)^27^, Sharon McNamara (Seattle Children's Hospital)^27^, Deborah Meyers (Wake Forest University)^33^, Susan Millard (DeVos Children’s Butterworth Hospital, Spectrum Health Systems)^27^, Peter Mogayzel (Johns Hopkins University)^27^, Richard Moss (Stanford University)^27^, Tanda Murray (Johns Hopkins University)^30^, Dennis Nielson (University of California at San Francisco)^27^, Blakeslee Noyes (Cardinal Glennon Children’s Hospital)^27^, Wanda O'Neal (University of North Carolina Chapel Hill)^26^, David Orenstein (Children’s Hospital of Pittsburgh)^27^, Brian O'Sullivan (University of Massachusetts Memorial Health Care)^27^, Rhonda Pace (University of North Carolina Chapel Hill)^26^, Peter Pare (St. Paul’s Hospital)^30^, H. Worth Parker (Dartmouth-Hitchcock Medical Center, New Hampshire Cystic Fibrosis Center)^27^, Mary Ann Passero (Rhode Island Hospital)^27^, Elizabeth Perkett (Vanderbilt University)^27,^ Adrienne Prestridge (Children's Memorial Hospital)^27^, Nicholas M. Rafaels (Johns Hopkins University)^30^, Bonnie Ramsey (Seattle Children's Hospital, University of Washington)^27^, Elizabeth Regan (National Jewish Health)^24^, Clement Ren (University of Rochester)^27^, George Retsch-Bogart (University of North Carolina Chapel Hill)^27^, Michael Rock (University of Wisconsin Hospital and Clinics)^27^, Antony Rosen (Johns Hopkins University)^32^, Margaret Rosenfeld (Seattle Children's Hospital, University of Washington)^27^, Ingo Ruczinski (Johns Hopkins University School of Public Health)^13, 15, 30^, Andrew Sanford (University of British Columbia)^30^, David Schaeffer (Nemours Children's Clinic)^27^, Cindy Sell (University of North Carolina Chapel Hill)^26^, Daniel Sheehan (Children's Hospital of Buffalo)^27^, Edwin K. Silverman (Brigham and Women's Hospital, Harvard Medical School)^24, 30^, Don Sin (Children’s Medical Center of Dayton)^30^, Terry Spencer (Elliot Health System)^27^, Jackie Stonebraker (University of North Carolina Chapel Hill)^26^, Holly K. Tabor (Seattle Children's Hospital, University of Washington)^6, 10, 11, 17, 27^, Laurie Varlotta (St. Christopher’s Hospital for Children)^27^, Candelaria I. Vergara (Johns Hopkins University)^30^, Robert Weiss ^30^, Fred Wigley (Johns Hopkins University)^32^, Robert A. Wise (Johns Hopkins University)^30^, Fred A. Wright (University of North Carolina Chapel Hill)^26^, Mark M. Wurfel (University of Washington)^10, 14, 21^, Robert Zanni (Monmouth Medical Center)^27^, Fei Zou (University of North Carolina Chapel Hill)^26^

**SeattleGO**

Deborah A. Nickerson (University of Washington)^3, 4, 5, 7, 8, 9, 11, 15, 17, 18, 19^, Mark J. Rieder (University of Washington)^4, 11, 13, 15, 16, 17, 19^, Phil Green (University of Washington), Jay Shendure (University of Washington)^1, 8, 14, 16, 17^, Joshua M. Akey (University of Washington)^13, 14, 15^, Michael J. Bamshad (Seattle Children's Hospital, University of Washington)^4, 6, 7, 8, 10, 11, 13, 15, 17, 27^, Kristine L. Bucasas (Baylor College of Medicine)^15^, Carlos D. Bustamante (Stanford University School of Medicine)^3, 13, 15^, David R. Crosslin (University of Washington)^2, 9^, Evan E. Eichler (University of Washington)^19^, P. Keolu Fox^2^, Wenqing Fu (University of Washington)^13^, Adam Gordon (University of Washington)^11^, Simon Gravel (Stanford University School of Medicine)^13, 15^, Gail P. Jarvik (University of Washington)^9, 15^, Jill M. Johnsen (Puget Sound Blood Center, University of Washington)^2^, Mengyuan Kan (Baylor College of Medicine)^13^, Eimear E. Kenny (Stanford University School of Medicine)^3, 13, 15^, Jeffrey M. Kidd (Stanford University School of Medicine)^13, 15^, Fremiet Lara-Garduno (Baylor College of Medicine)^15^, Suzanne M. Leal (Baylor College of Medicine)^1, 13, 15, 16, 17, 19, 20^, Dajiang J. Liu (Baylor College of Medicine)^13, 15^, Sean McGee (University of Washington)^13, 15, 19^, Timothy D. O’Connor (University of Washington)^13^, Bryan Paeper (University of Washington)^16^, Peggy D. Robertson (University of Washington)^4^, Joshua D. Smith (University of Washington)^4, 16, 19^, Jeffrey C. Staples (University of Washington), Jacob A. Tennessen (University of Washington)^13^, Emily H. Turner (University of Washington)^4, 16^, Gao Wang (Baylor College of Medicine)^1,13,20^, Qian Yi (University of Washington)^4^

**WHISP**

Rebecca Jackson (Ohio State University)^1, 2, 4, 5, 8, 12, 14, 15, 17, 18, 20, 34^, Kari North (University of North Carolina Chapel Hill)^1, 3, 9, 10, 13, 15, 17, 34,^ Ulrike Peters (Fred Hutchinson Cancer Research Center)^1, 3, 11, 12, 13, 15, 17, 18, 34^, Christopher S. Carlson (Fred Hutchinson Cancer Research Center, University of Washington)^1, 2, 3, 5, 12, 13, 14, 15, 16, 17, 18, 19, 34^, Garnet Anderson (Fred Hutchinson Cancer Research Center)^34^, Hoda Anton-Culver (University of California at Irvine)^34^, Themistocles L. Assimes (Stanford University School of Medicine)^5, 9, 11, 34^, Paul L. Auer (Fred Hutchinson Cancer Research Center)^1, 2, 3^, ^5, 11, 12, 13, 15, 16, 18, 34^, Shirley Beresford (Fred Hutchinson Cancer Research Center)^34^, Chris Bizon (University of North Carolina Chapel Hill)^3, 9, 13, 15, 34^, Henry Black (Rush Medical Center)^34^, Robert Brunner (University of Nevada)^34^, Robert Brzyski (University of Texas Health Science Center San Antonio)^34^, Dale Burwen (National Heart, Lung, and Blood Institute WHI Project Office)^34^, Bette Caan (Kaiser Permanente Division of Research, Oakland, CA)^34^, Cara L. Carty (Fred Hutchinson Cancer Research Center)^18, 34^, Rowan Chlebowski (Los Angeles Biomedical Research Institute)^34^, Steven Cummings (University of California at San Francisco)^34^, J. David Curb* (University of Hawaii)^9, 18, 34^, Charles B. Eaton (Brown University, Memorial Hospital of Rhode Island)^12, 34^, Leslie Ford (National Heart, Lung, and Blood Institute, National Heart, Lung, and Blood Institute WHI Project Office)^34^, Nora Franceschini (University of North Carolina Chapel Hill)^2, 3, 9, 10, 15, 34^, Stephanie M. Fullerton (University of Washington)^6, 11, 34^, Margery Gass (University of Cincinnati)^34^, Nancy Geller (National Heart, Lung, and Blood Institute WHI Project Office)^34^, Gerardo Heiss (University of North Carolina Chapel Hill)^5, 34^, Barbara V. Howard (Howard University, MedStar Research Institute)34, Li Hsu (Fred Hutchinson Cancer Research Center)^1, 13, 15, 18, 34^, Carolyn M. Hutter (Fred Hutchinson Cancer Research Center)^13, 15, 18, 34^, John Ioannidis (Stanford University School of Medicine)^11, 34^, Shuo Jiao (Fred Hutchinson Cancer Research Center)^34^, Karen C. Johnson (University of Tennessee Health Science Center)^3, 34^, Charles Kooperberg (Fred Hutchinson Cancer Research Center)^1, 5, 9, 13, 14, 15, 17, 18, 34^, Lewis Kuller (University of Pittsburgh)^34^, Andrea LaCroix (Fred Hutchinson Cancer Research Center)^34^, Kamakshi Lakshminarayan (University of Minnesota)^18, 34^, Dorothy Lane (State University of New York at Stony Brook)^34^, Ethan M. Lange (University of North Carolina Chapel Hill)^3, 5, 9, 13, 34^, Leslie A. Lange (University of North Carolina Chapel Hill)^1, 2, 3, 5, 9, 12, 13, 15, 17, 18, 20, 25, 34^, Norman Lasser (University of Medicine and Dentistry of New Jersey)^34^, Erin LeBlanc (Kaiser Permanente Center for Health Research, Portland, OR)^34^, Cora E. Lewis (University of Alabama at Birmingham)^25,34^, Kuo-Ping Li (University of North Carolina Chapel Hill)^9, 34^, Marian Limacher (University of Florida)^34^, Dan-Yu Lin (University of North Carolina Chapel Hill)^1, 3, 9, 13, 15, 34^, Benjamin A. Logsdon (Fred Hutchinson Cancer Research Center)^2, 34^, Shari Ludlam (National Heart, Lung, and Blood Institute WHI Project Office)^34^, JoAnn E. Manson (Brigham and Women's Hospital, Harvard School of Public Health)^34^, Karen Margolis (University of Minnesota)^34^, Lisa Martin (George Washington University Medical Center)^9, 34^, Joan McGowan (National Heart, Lung, and Blood Institute WHI Project Office)^34^, Keri L. Monda (Amgen, Inc.)^1,^ ^15, 34^, Jane Morley Kotchen (Medical College of Wisconsin)^34^, Lauren Nathan (University of California Los Angeles)^34^, Judith Ockene (Fallon Clinic, University of Massachusetts)^34^, Mary Jo O'Sullivan (University of Miami)^34^, Lawrence S. Phillips (Emory University)^34^, Ross L. Prentice (Fred Hutchinson Cancer Research Center)^34^, Alexander P. Reiner (Fred Hutchinson Cancer Research Center, University of Washington)^1, 2, 3, 5, 9, 11, 12, 13, 14, 15, 20, 25, 34^, John Robbins (University of California at Davis)^34^, Jennifer G. Robinson (University of Iowa)^9, 11, 18, 34^, Jacques E. Rossouw (National Heart, Lung, and Blood Institute, National Heart, Lung, and Blood Institute WHI Project Office)^5, 14, 17, 20, 34^, Haleh Sangi-Haghpeykar (Baylor College of Medicine)^34^, Gloria E. Sarto (University of Wisconsin)^34^, Sally Shumaker (Wake Forest University)^34^, Michael S. Simon (Wayne State University)^34^, Marcia L. Stefanick (Stanford University School of Medicine)^34^, Evan Stein (Medical Research Labs)^34^, Hua Tang (Stanford University)^2, 34^, Kira C. Taylor (University of Louisville)^1, 3, 13, 15, 20, 34^, Cynthia A. Thomson (University of Arizona)^34^, Timothy A. Thornton (University of Washington)^13, 15, 18, 34^, Linda Van Horn (Northwestern University)^34^, Mara Vitolins (Wake Forest University)^34^, Jean Wactawski-Wende (University of Buffalo)^34^, Robert Wallace (University of Iowa)^2, 34^, Sylvia Wassertheil-Smoller (Boston University)^18, 34^, Donglin Zeng (University of North Carolina Chapel Hill)^9, 34^

*deceased

**NHLBI GO ESP Project Team**

Deborah Applebaum-Bowden (National Heart, Lung, and Blood Institute)^4, 7, 12, 17^, Michael Feolo (National Center for Biotechnology Information)^12^, Weiniu Gan (National Heart, Lung, and Blood Institute)^7, 8, 16, 17^, Dina N. Paltoo (National Heart, Lung, and Blood Institute)^4, 6, 11, 17^, Jacques E. Rossouw (National Heart, Lung, and Blood Institute, National Heart, Lung, and Blood Institute WHI Project Office)^5, 14, 17, 20, 34^, Phyliss Sholinsky (National Heart, Lung, and Blood Institute)^4, 12, 17^, Anne Sturcke (National Center for Biotechnology Information)^12^

**ESP Groups**

^1^Anthropometry Project Team, ^2^Blood Count/Hematology Project Team, ^3^Blood Pressure Project Team, ^4^Data Flow Working Group, ^5^Early MI Project Team, ^6^ELSI Working Group, ^7^Executive Committee, ^8^Family Study Project Team, ^9^Lipids Project Team, ^10^Lung Project Team, ^11^Personal Genomics Project Team, ^12^Phenotype and Harmonization Working Group, ^13^Population Genetics and Statistical Analysis Working Group, ^14^Publications and Presentations Working Group, ^15^Quantitative Analysis Ad Hoc Task Group, ^16^Sequencing and Genotyping Working Group, ^17^Steering Committee, ^18^Stroke Project Team, ^19^Structural Variation Working Group, ^20^Subclinical/Quantitative Project Team

**ESP Cohorts**

^21^Acute Lung Injury (ALI), ^22^Atherosclerosis Risk in Communities (ARIC), ^23^Cardiovascular Health Study (CHS), ^24^Chronic Obstructive Pulmonary Disease (COPDGene), ^25^Coronary Artery Risk Development in Young Adults (CARDIA), ^26^Cystic Fibrosis (CF), ^27^Early Pseudomonas Infection Control (EPIC), ^28^Framingham Heart Study (FHS), ^29^Jackson Heart Study (JHS), ^30^Lung Health Study (LHS), ^31^Multi-Ethnic Study of Atherosclerosis (MESA), ^32^Pulmonary Arterial Hypertension (PAH), ^33^Severe Asthma Research Program (SARP), ^34^Women's Health Initiative (WHI)

**The GoT2D Consortium**

Gonçalo Abecasis^1^, Vineeta Agarwala^2^, Peter Algren^3^, David Altshuler^2,4,5,6,7,8,9^, Martin Hrabé de Angelis^10^, Eric Banks^2^, Richard Bergman^11^, Thomas Blackwell^1^, Michael Boehnke^1^, Lori Bonnycastle^12^, David Buck^13^, Martijn van de Bunt^14^, Noël Burtt^2^, Peter Chines^12^, Francis Collins^12^, Mark DePristo^2^, Peter Donnelly^13^, Timothy Fennell^2^, Jason Flannick^2,5^, Pierre Fontanillas^2^, Timothy Frayling^15^, Christian Fuchsberger^1^, Stacey Gabriel^2^, Kyle Gaulton^13^, Christian Gieger^10^, Harald Grallert^10^, Todd Green^2^, Leif Groop^3^, Christopher Hartl^2^, Andrew Hattersley^15^, Bryan Howie^16^, Cornelia Huth^10^, Jeroen Huyghe^1^, Bo Isomaa^17,18^, Anne Jackson^19^, Goo Jun^1^, Hyun Min Kang^1^, Jasmina Kravic^3^, Jennifer Kriebel^10^, Ashish Kumar^13^, Phoenix Kwan^1^, Claes Ladenvall^3^, Cecilia Lindgren^13^, Adam Locke^1^, Gerton Lunter^13^, Clement Ma^1^, Anubha Mahajan^13^, Alisa Manning^2^, Mark McCarthy^13,14,20^ Gil McVean^13^, Christa Meisinger^10^, Thomas Meitinger^21^, Karen Mohlke^22^, Andrew Morris^13,23^, Loukas Moutsianas^13^, Martina Müller-Nurasyid^10^, Pål Njølstad^24,25^, Richard Pearson^13^, John Perry^13^, Annette Peters^10^, Ryan Poplin^2^, Inga Prokopenko^13,26^, Wolfgang Rathmann^10^, William Rayner^13^, Janina Ried^10^, Manuel Rivas^13^, Neil Robertson^13^, Laura Scott^1^, Khalid Shakir^2^, Xueling Sim^1^, Kerrin Small^27^, Timothy Spector^27^, Michael Stitzel^28^, Konstantin Strauch^10^, Heather Stringham^1^, Tim Strom^21^, Adrian Tan^1^, Tanya Teslovich^1^, Tiinamaija Toumi^17,18^, Jaakko Tuomilehto^29^

**The T2D-GENES Consortium**

Gonçalo Abecasis^1^, Marcio Almeida^30^, David Altshuler^2,4,5,6,7,8,9^, Jennifer Asimit^31^, Gil Atzmon^32^, Mathew Barber^16^, Nir Barzilai^32^, Nicola Beer^14^, Graeme Bell^16,33^, Jennifer Below^34^, Tom Blackwell^1^, John Blangero^30^, Michael Boehnke^1^, Donald Bowden^35^, Noël Burtt^2^, John Chambers^36,37,38^, Han Chen^39^, Peng Chen^40^, Peter Chines^12^, Sungkyoung Choi^41^, Claire Churchhouse^2^, Pablo Cingolani^42^, Belinda Cornes^43^, Nancy Cox^16,33^, Aaron Day-Williams^31^, Ravindranath Duggirala^30^, Josée Dupuis^39^, Thomas Dyer^30^, Shuang Feng^1^, Juan Fernandez-Tajes^13^, Teresa Ferreira^13^, Tasha Fingerlin^44^, Jason Flannick^2,5^, Jose Florez^2,5,6^, Pierre Fontanillas^2^, Timothy Frayling^15^, Christian Fuchsberger^1^, Eric Gamazon^33^, Kyle Gaulton^13^, Saurabh Ghosh^45^, Benjamin Glaser^46^, Anna Gloyn^14^, Robert Grossman^33,47^, Jason Grundstad^47^, Craig Hanis^34^, Allison Heath^47^, Heather Highland^34^, Momoko Hirokoshi^13^, Ik-Soo Huh^41^, Jeroen Huyghe^1^, Kamran Ikram^48,43,49,50^, Kathleen Jablonski^51^, Young Jin Kim^52^, Goo Jun^25^, Norihiro Kato^53^, Jayoun Kim^41^, Ryan King^54^, Jaspal Kooner^37,38,55^, Min-Seok Kwon^41^, Hae Kyung Im^54^, Markku Laakso^56^, Kevin Koi-Yau Lam^40^, Jaehoon Lee^41^, Selyeong Lee^41^, Sungyoung Lee^47^, Donna Lehman^57^, Heng Li^2^, Cecilia Lindgren^13^, Xuanyao Liu^40,58^, Oren Livne^16^, Adam Locke^1^, Anubha Mahajan^13^, Julian Maller^13,59^, Alisa Manning^2^, Taylor Maxwell^34^, Alexander Mazur^60^, Mark McCarthy^13,14,20^, James Meigs^6,61^, Byungju Min^41^, Karen Mohlke^22^, Andrew Morris^13,23^, Solomon Musani^62^, Yoshihiko Nagai^60^, Maggie Ng^35^, Dan Nicolae^16,33,12^, Sohee Oh^41^, Nicholette Palmer^35^, Taesung Park^41^, Toni Pollin^63^, Inga Prokopenko^13,26^, David Reich^2,4^, Manuel Rivas^13^, Laura Scott^1^, Mark Seielstad^64^, Yoon Shin Cho^65^, E-Shyong Tai^48,40,66^, Xueling Sim1, Robert Sladek^60,67^, Philip Smith^68^, Ioanna Tachmazidou^31^, Tanya Teslovich^1^, Jason Torres^16,33^, Vasily Trubetskoy^16,33^, Sara Willems^69^, Amy Williams^2,4^, James Wilson^70^, Steven Wiltshire^71^, Sungho Won^72^, Andrew Wood^15^, Wang Xu^66^, Yik Ying Teo^73,74,75,58,76^, Joon Yoon^41^, Jong-Young Lee^77^, Matthew Zawistowski^1^, Eleftheria Zeggini^31^, Weihua Zhang^36^, Sebastian Zöllner^1,78^

**Affiliations**

1. Department of Biostatistics, Center for Statistical Genetics, University of Michigan, Ann Arbor, MI,

USA

2. Program in Medical and Population Genetics, Broad Institute of Harvard and MIT, Cambridge, Massachusetts

02142, USA

3. Department of Clinical Sciences, Diabetes and Endocrinology, Clinical Research Centre, Lund University,

Malmö, Sweden

4. Department of Genetics, Harvard Medical School, Boston, Massachusetts 02115, USA

5. Center for Human Genetic Research and Diabetes Research Center (Diabetes Unit), Massachusetts

General Hospital, Boston, MA, USA

6. Department of Medicine, Harvard Medical School, Boston, MA, USA

7. Center for Human Genetic Research, Massachusetts General Hospital, Boston, MA, USA

8. Department of Molecular Biology, Harvard Medical School, Boston, MA, USA

9. Department of Biology, Massachusetts Institute of Technology, Cambridge, MA, USA

10. Institute of Genetic Epidemiology, Helmholtz Zentrum München, Neuherberg, Germany

11. Department of Preventive Medicine, Keck School of Medicine, University of Southern California, Los

Angeles, CA, USA

12. National Human Genome Research Institute, National Institutes of Health, Bethesda, MD, USA

13. Wellcome Trust Centre for Human Genetics, University of Oxford, Oxford, UK

14. Oxford Centre for Diabetes, Endocrinology and Metabolism, University of Oxford, Oxford, UK

15. Genetics of Complex Traits, University of Exeter Medical School, Exeter, UK

16. Department of Human Genetics, University of Chicago, Chicago, IL, USA

17. Department of Medicine, Helsinki University Hospital, Helsinki, Finland

18. Folkhälsan Research Center, Helsinki, Finland

19. Department of Pathology, University of Michigan, Ann Arbor, MI, USA

20. Oxford NIHR Biomedical Research Centre, Churchill Hospital, Headington, UK

21. Human Genetics, Helmholtz Zentrum München, Neuherberg, Germany

22. Department of Genetics, University of North Carolina-Chapel Hill, Chapel Hill, NC, USA

23. Department of Biostatistics, University of Liverpool, Liverpool, UK

24. Department of Clinical Science, University of Bergen, Bergen, Norway

25. Department of Pediatrics, Haukeland University Hospital, Bergen, Norway

26. Department of Medical Sciences, Molecular Epidemiology and Science for Life Laboratory, Uppsala

University, Uppsala, Sweden

27. Kings College of London, Department of Twin Research and Genetic Epidemiology, London, UK

28. Department of Biochemistry and Molecular Biology, Pennsylvania State University, State College,

PA

29. Department of Public Health, University of Helsinki, Helsinki, Finland

30. Department of Genetics, Texas Biomedical Research Institute, San Antonio, TX, USA

31. Wellcome Trust Sanger Institute, Hinxton, Cambridge, UK

32. Department of Medicine, Department of Genetics, Albert Einstein College of Medicine, NY, USA

33. Department of Medicine, University of Chicago, Chicago, IL, USA

34. Human Genetics Center, University of Texas Health Science Center at Houston, Houston, TX, USA

35. Center for Genomics and Personalized Medicine Research, Center for Diabetes Research, Department

of Biochemistry, Department of Internal Medicine, Wake Forest School of Medicine, Winston-

Salem, NC, USA

36. Department of Epidemiology and Biostatistics, Imperial College London, London, UK

37. Imperial College Healthcare NHS Trust, London, UK

38. Ealing Hospital National Health Service (NHS) Trust, Middlesex, UK

39. Department of Biostatistics, Boston University School of Public Health, Boston, MA, USA

40. Saw Swee Hock School of Public Health, National University of Singapore, Singapore, Singapore

41. Seoul National University, Seoul, South Korea

42. McGill Centre for Bioinformatics, McGill University, MontrÃl’al, Quebec, Canada

43. Singapore Eye Research Institute, Singapore National Eye Centre, Singapore, Singapore

44. Department of Epidemiology, Colorado School of Public Health, Aurora, CO, USA

45. Department of Textile Technology, Indian Institute of Technology Delhi, New Delhi, India

46. Department of Endocrinology, Hadassah Medical Center, Kiryat Hadassah, Jerusalem, Israel

47. Institute for Genomics and Systems Biology, University of Chicago, Chicago, IL, USA

48. Duke-National University of Singapore Graduate Medical School, Singapore, Singapore

49. Department of Ophthalmology, National University of Singapore and National University Health System,

Singapore, Singapore

50. Department of Ophthalmology, Erasmus Medical Center, Rotterdam, the Netherlands

51. The Biostatistics Center, George Washington University, Rockville, MD, USA

52. Department of Neurology, Konkuk University School of Medicine, Seoul, South Korea

53. Department of Gene Diagnostics and Therapeutics, Research Institute, National Center for Global

Health and Medicine, Tokyo, Japan

54. Department of Health Studies, University of Chicago, Chicago, IL, USA

55. National Heart and Lung Institute (NHLI), Imperial College London, Hammersmith Hospital, London,

UK

56. Department of Medicine, University of Eastern Finland, Kuopio Campus and Kuopio University Hospital,

Kuopio, Finland

57. Division of Clinical Epidemiology, Department of Medicine, University of Texas Health Science Center

at San Antonio, San Antonio, TX, USA

58. Graduate School for Integrative Science and Engineering, National University of Singapore, Singapore,

Singapore

59. Department of Statistics, University of Oxford, Oxford, UK

60. McGill University, Montréal, Québec, Canada

61. General Medicine Division, Massachusetts General Hospital, Boston, MA, USA

62. Department of Medicine, University of Mississippi Medical Center, Jackson, MS, USA

63. Department of Medicine, Program for Personalized and Genomic Medicine, University of Maryland

School of Medicine, Baltimore, MD, USA

64. University of California San Francisco, San Francisco, CA, USA

65. Department of Biomedical Science, Hallym University, Chuncheon, Gangwon-do, South Korea

66. Department of Medicine, National University of Singapore, National University Health System, Singapore,

Singapore

67. Department of Medicine, Royal Victoria Hospital, Montréal, Québec, Canada

68. National Institute of Diabetes and Digestive and Kidney Disease, National Institutes of Health,

Bethesda, MD, USA

69. Department of Genetic Epidemiology, Erasmus Medical Center, Rotterdam, the Netherlands

70. Department of Physiology and Biophysics, University of Mississippi Medical Center, Jackson, MS,

USA

71. Centre for Medical Research, Western Australian Institute for Medical Research, The University of

Western Australia, Nedlands, WA, Australia

72. Chung-Ang University, Seoul, South Korea

73. Department of Epidemiology and Public Health, National University of Singapore, Singapore, Singapore

74. Centre for Molecular Epidemiology, National University of Singapore, Singapore, Singapore

75. Genome Institute of Singapore, Agency for Science, Technology and Research, Singapore, Singapore

76. Department of Statistics and Applied Probability, National University of Singapore, Singapore, Singapore

77. Center for Genome Science, Korea National Institute of Health, Osong Health Technology Administration

Complex, Chungcheongbuk-do, South Korea

78. Department of Psychiatry, University of Michigan, Ann Arbor, MI, USA

**Genetic Investigation of ANthropometric Traits (GIANT) Consortium.**

Andrew R Wood^1^, Tonu Esko^2,3,4,5^, Jian Yang^6,7^, Sailaja Vedantam^3,4^, Tune H Pers^3,4,5,8^, Stefan Gustafsson^9,10^, Audrey Y Chu^11^, Karol Estrada^4,12,13^, Jian'an Luan^14^, Zoltán Kutalik^15,16,17^, Najaf Amin^18^, Martin L Buchkovich^19^, Damien C Croteau-Chonka^19,20^, Felix R Day^14^, Yanan Duan^21^, Tove Fall^9,10,22^, Rudolf Fehrmann^23^, Teresa Ferreira^24^, Anne U Jackson^25^, Juha Karjalainen^23^, Ken Sin Lo^26^, Adam E Locke^25^, Reedik Mägi^2,24^, Evelin Mihailov^2,27^, Eleonora Porcu^28^, Joshua C Randall^24,29^, André Scherag^30,31^, Anna AE Vinkhuyzen^6^, Harm-Jan Westra^23^, Thomas W Winkler^32^, Tsegaselassie Workalemahu^33^, Jing Hua Zhao^14^, Devin Absher^34^, Eva Albrecht^35^, Denise Anderson^36^, Jeffrey Baron^37^, Marian Beekman^38,39^, Ayse Demirkan^18,40^, Georg B Ehret^41,42^, Bjarke Feenstra^43^, Mary F Feitosa^44^, Krista Fischer^2^, Ross M Fraser^45^, Anuj Goel^24,46^, Jian Gong^47^, Anne E Justice^48^, Stavroula Kanoni^49^, Marcus E Kleber^50,51^, Kati Kristiansson^52^, Unhee Lim^53^, Vaneet Lotay^54^, Julian C Lui^37^, Massimo Mangino^55^, Irene Mateo Leach^56^, Carolina Medina-Gomez^12,57,58^, Michael A Nalls^59^, Dale R Nyholt^60^, Cameron D Palmer^3,4^, Dorota Pasko^1^, Sonali Pechlivanis^30^, Inga Prokopenko^24,61,62^, Janina S Ried^35^, Stephan Ripke^13,63^, Dmitry Shungin^64,65,66^, Alena Stancáková^67^, Rona J Strawbridge^68^, Yun Ju Sung^69^, Toshiko Tanaka^70^, Alexander Teumer^71^, Stella Trompet^72,73^, Sander W van der Laan^74^, Jessica van Setten^75^, Jana V Van Vliet-Ostaptchouk^76^, Zhaoming Wang^77,78,79,80^, Loïc Yengo^81,82,83^, Weihua Zhang^84,85^, Uzma Afzal^84,85^, Johan Ärnlöv^9,10,86^, Gillian M Arscott^87^, Stefania Bandinelli^88^, Amy Barrett^61^, Claire Bellis^89^, Amanda J Bennett^61^, Christian Berne^90^, Matthias Blüher^91,92^, Jennifer L Bolton^45^, Yvonne Böttcher^91^, Heather A Boyd^43^, Marcel Bruinenberg^93^, Brendan M Buckley^94^, Steven Buyske^95,96^, Ida H Caspersen^97^, Peter S Chines^98^, Robert Clarke^99^, Simone Claudi-Boehm^100^, Matthew Cooper^36^, E Warwick Daw^44^, Pim A De Jong^101^, Joris Deelen^38,39^, Graciela Delgado^50^, Josh C Denny^102^, Rosalie Dhonukshe-Rutten^103^, Maria Dimitriou^104^, Alex SF Doney^105^, Marcus Dörr^77,106^, Niina Eklund^52,107^, Elodie Eury^81,82,83^, Lasse Folkersen^68^, Melissa E Garcia^108^, Frank Geller^43^, Vilmantas Giedraitis^109^, Alan S Go^110^, Harald Grallert^35,111,112^, Tanja B Grammer^50^, Jürgen Gräßler^113^, Henrik Grönberg^22^, Lisette C.P.G.M. de Groot^103^, Christopher J Groves^61^, Jeffrey Haessler^47^, Per Hall^22^, Toomas Haller^2^, Goran Hallmans^114^, Anke Hannemann^78^, Catharina A Hartman^115^, Maija Hassinen^116^, Caroline Hayward^117^, Nancy L Heard-Costa^118,119^, Quinta Helmer^38,120,121^, Gibran Hemani^6,7^, Anjali K Henders^60^, Hans L Hillege^56,122^, Mark A Hlatky^123^, Wolfgang Hoffmann^77,124^, Per Hoffmann^125,126,127^, Oddgeir Holmen^128^, Jeanine J Houwing-Duistermaat^38,120^, Thomas Illig^111,129^, Aaron Isaacs^18,130^, Alan L James^131,132^, Janina Jeff^54^, Berit Johansen^97^, Åsa Johansson^133^, Jennifer Jolley^134,135^, Thorhildur Juliusdottir^24^, Juhani Junttila^136^, Abel N Kho^137^, Leena Kinnunen^52^, Norman Klopp^111,129^, Thomas Kocher^138^, Wolfgang Kratzer^139^, Peter Lichtner^140^, Lars Lind^141^, Jaana Lindström^52^, Stéphane Lobbens^81,82,83^, Mattias Lorentzon^142^, Yingchang Lu^54,143^, Valeriya Lyssenko^144^, Patrik KE Magnusson^22^, Anubha Mahajan^24^, Marc Maillard^145^, Wendy L McArdle^146^, Colin A McKenzie^147^, Stela McLachlan^45^, Paul J McLaren^148,149^, Cristina Menni^55^, Sigrun Merger^100^, Lili Milani^2^, Alireza Moayyeri^55^, Keri L Monda^48,150^, Mario A Morken^98^, Gabriele Müller^151^, Martina Müller-Nurasyid^35,152,153,154^, Arthur W Musk^155^, Narisu Narisu^98^, Matthias Nauck^77,78^, Ilja M Nolte^122^, Markus M Nöthen^126,127^, Laticia Oozageer^84^, Stefan Pilz^156,157^, Nigel W Rayner^24,29,61^, Frida Renstrom^64^, Neil R ^Robertson24,61^, Lynda M Rose^11^, Ronan Roussel^158,159,160^, Serena Sanna^28^, Hubert Scharnagl^161^, Salome Scholtens^122,^ Fredrick R Schumacher^162^, Heribert Schunkert^154,163^, Robert A Scott^14^, Joban Sehmi^84,85^, Thomas Seufferlein^139^, Jianxin Shi^164^, Karri Silventoinen^165^, Johannes H Smit^166,167^, Albert Vernon Smith^168,169^, Joanna Smolonska^23,122^, Alice V Stanton^170^, Kathleen Stirrups^29,49^, David J Stott^171^, Heather M Stringham^25^, Johan Sundström^141^, Morris A Swertz^23^, Ann-Christine Syvänen^9,172^, Bamidele O Tayo^173^, Gudmar Thorleifsson^174^, Jonathan P Tyrer^175^, Suzanne van Dijk^12^, Natasja M van Schoor^156^, Nathalie van der Velde^12,176^, Diana van Heemst^38,73^, Floor VA van Oort^177^, Sita H Vermeulen^178,179^, Niek Verweij^56^, Judith M Vonk^122^, Lindsay L Waite^34^, Melanie Waldenberger^111^, Roman Wennauer^180^, Lynne R Wilkens^53^, Christina Willenborg^181,182^, Tom Wilsgaard^183^, Mary K Wojczynski^44^, Andrew Wong^184^, Alan F Wright^117^, Qunyuan Zhang^44^, Dominique Arveiler^185^, Stephan JL Bakker^186^, John Beilby^87,187^, Richard N Bergman^188^, Sven Bergmann^16,17^, Reiner Biffar^189^, John Blangero^89^, Dorret I Boomsma^190^, Stefan R Bornstein^113^, Pascal Bovet^191,192^, Paolo Brambilla^193^, Morris J Brown^194^, Harry Campbell^45^, Mark J Caulfield^195^, Aravinda Chakravarti^41^, Rory Collins^99^, Francis S Collins^98^, Dana C Crawford^196,197^, L Adrienne Cupples^118,198^, John Danesh^199^, Ulf de Faire^200^, Hester M den Ruijter^74,201^, Raimund Erbel^202^, Jeanette Erdmann^181,182^, Johan G Eriksson^52,203,204^, Martin Farrall^24,46^, Ele Ferrannini^205,206^, Jean Ferrières^207^, Ian Ford^208^, Nita G Forouhi^14^, Terrence Forrester^147^, Ron T Gansevoort^186^, Pablo V Gejman^209^, Christian Gieger^35^, Alain Golay^210^, Omri Gottesman^54^, Vilmundur Gudnason^168,169^, Ulf Gyllensten^133^, David W Haas^211^, Alistair S Hall^212^, Tamara B Harris^108^, Andrew T Hattersley^213^, Andrew C Heath^214^, Christian Hengstenberg^154,163^, Andrew A Hicks^215,216^, Lucia A Hindorff^217^, Aroon D Hingorani^218^, Albert Hofman^57,58^, G Kees Hovingh^219^, Steve E Humphries^220^, Steven C Hunt^221^, Elina Hypponen^222,223,224^, Kevin B Jacobs^79,80^, Marjo-Riitta Jarvelin^85,225,226,227,228,229^, Pekka Jousilahti^52^, Antti M Jula^52^, Jaakko Kaprio^52,107,230^, John JP Kastelein^219^, Manfred Kayser^57,231^, Frank Kee^232^, Sirkka M Keinanen-Kiukaanniemi^233,234^, Lambertus A Kiemeney^178,235^, Jaspal S Kooner^84,236,237^, Charles Kooperberg^47^, Seppo Koskinen^52^, Peter Kovacs^91,92^, Aldi T Kraja^44^, Meena Kumari^238^, Johanna Kuusisto^239^, Timo A Lakka^116,240,241^, Claudia Langenberg^14,238^, Loic Le Marchand^53^, Terho Lehtimäki^242^, Sara Lupoli^243,244^, Pamela AF Madden^214^, Satu Männistö^52^, Paolo Manunta^245,246^, André Marette^247,248^, Tara C Matise^96^, Barbara McKnight^249^, Thomas Meitinger^154^, Frans L Moll^250^, Grant W Montgomery^60^, Andrew D Morris^105^, Andrew P Morris^2,24,251^, Jeffrey C Murray^252^, Mari Nelis^2^, Claes Ohlsson^142^, Albertine J Oldehinkel^115^, Ken K Ong^14,184^, Willem H Ouwehand^134,135^, Gerard Pasterkamp^74^, Annette Peters^111,154,253^, Peter P Pramstaller^215,216,254^, Jackie F Price^45^, Lu Qi^20,255^, Olli T Raitakari^256,257^, Tuomo Rankinen^258^, DC Rao^44,69,214^, Treva K Rice^69,214^, Marylyn Ritchie^259^, Igor Rudan^45,260^, Veikko Salomaa^52^, Nilesh J Samani^261,262^, Jouko Saramies^263^, Mark A Sarzynski^258^, Peter EH Schwarz^113,264^, Sylvain Sebert^229^, Peter Sever^265^, Alan R Shuldiner^266,267^, Juha Sinisalo^268^, Valgerdur Steinthorsdottir^174^, Ronald P Stolk^122^, Jean-Claude Tardif^26,269^, Anke Tönjes^91,92^, Angelo Tremblay^270^, Elena Tremoli^271^, Jarmo Virtamo^52^, Marie-Claude Vohl^248,272^, The electronic medical records and genomics (eMERGE) consortium^273^, The MIGen Consortium^274,275^, The PAGE Consortium^275,276^, The LifeLines Cohort Study^275,277^, Philippe Amouyel^278^, Folkert W Asselbergs^218,279,280^, Themistocles L Assimes^123^, Murielle Bochud^191,192^, Bernhard O Boehm^100,281^, Eric Boerwinkle^282^, Erwin P Bottinger^54^, Claude Bouchard^258^, Stéphane Cauchi^81,82,83^, John C Chambers^84,85,236^, Stephen J Chanock^79^, Richard S Cooper^173^, Paul IW de Bakker^75,283,284^, George Dedoussis^104^, Luigi Ferrucci^70^, Paul W Franks^64,65,255^, Philippe Froguel^62,81,82,83^, Leif C Groop^107,285^, Christopher A Haiman^162^, Anders Hamsten^68^, M Geoffrey Hayes^137^, Jennie Hui^87,187,222^, David J. Hunter^20,255,286^, Kristian Hveem^128^, J Wouter Jukema^72,280,287^, Robert C Kaplan^288^, Mika Kivimaki^238^, Diana Kuh^184^, Markku Laakso^239^, Yongmei Liu^289^, Nicholas G Martin^60^, Winfried März^50,161,290^, Mads Melbye^43,123^, Susanne Moebus^30^, Patricia B Munroe^195,^

Inger Njølstad^183^, Ben A Oostra^18,130,291^, Colin NA Palmer^105^, Nancy L Pedersen^22^, Markus Perola^2,52,107^, Louis Pérusse^248,270^, Ulrike Peters^47^, Joseph E Powell^6,7^, Chris Power^224^, Thomas Quertermous^123^, Rainer Rauramaa^116,241^, Eva Reinmaa^2^, Paul M Ridker^11,292^, Fernando Rivadeneira^12,57,58^, Jerome I Rotter^293^, Timo E Saaristo^294,295^, Danish Saleheen^199,296,297^, David Schlessinger^298^, P Eline Slagboom^38,39^, Harold Snieder^122^, Tim D Spector^55^, Konstantin Strauch^35,153^, Michael Stumvoll^91,92^, Jaakko Tuomilehto^52,299,300,301^, Matti Uusitupa^302,303^, Pim van der Harst^23,56,280^, Henry Völzke^77,124^, Mark Walker^304^, Nicholas J Wareham^14^, Hugh Watkins^24,46^, H-Erich Wichmann^305,306,307^,, James F Wilson^45^, Pieter Zanen^308^, Panos Deloukas^29,49,309^, Iris M Heid^32,35^, Cecilia M Lindgren^4,24^, Karen L Mohlke^19^, Elizabeth K Speliotes^310^, Unnur Thorsteinsdottir^174,311^, Inês Barroso^29,312,313^, Caroline S Fox^118^, Kari E North^48,314^, David P Strachan^315^, Jacques S. Beckmann^16,17,316^, Sonja I Berndt^79^, Michael Boehnke^25^, Ingrid B Borecki^44^, Mark I McCarthy^24,61,317^, Andres Metspalu^2,27^, Kari Stefansson174,311, André G Uitterlinden^12,57,58^, Cornelia M van Duijn^18,57,58,130^, Lude Franke^23^, Cristen J Willer^318,319,320^, Alkes L. Price^4,286,321^, Guillaume Lettre^26,269^, Ruth JF Loos^14,54,143,322^, Michael N Weedon^1^, Erik Ingelsson^9,10,24^, Jeffrey R O'Connell^266^, Goncalo R Abecasis^25^, Daniel I Chasman^11,292^, Michael E Goddard^323,324^, Peter M Visscher^6,7^, Joel N Hirschhorn^3,4,5^, Timothy M Frayling^1^

**Affiliations**

1. Genetics of Complex Traits, University of Exeter Medical School, University of Exeter, Exeter EX1 2LU, UK

2. Estonian Genome Center, University of Tartu, Tartu 51010, Estonia

3. Division of Endocrinology, Genetics and Basic and Translational Obesity Research, Boston Children's Hospital, Boston, MA 02115, USA

4. Broad Institute of the Massachusetts Institute of Technology and Harvard University, Cambridge 02142, MA, USA

5. Department of Genetics, Harvard Medical School, Boston, MA 02115, USA

6. Queensland Brain Institute, The University of Queensland, Brisbane 4072, Australia

7. The University of Queensland Diamantina Institute, The Translation Research Institute, Brisbane 4012, Australia

8. Center for Biological Sequence Analysis, Department of Systems Biology, Technical University of Denmark, Lyngby 2800, Denmark

9. Science for Life Laboratory, Uppsala University, Uppsala 75185, Sweden

10. Department of Medical Sciences, Molecular Epidemiology, Uppsala University, Uppsala 75185, Sweden

11. Division of Preventive Medicine, Brigham and Women's Hospital, Boston, MA 02215, USA

12. Department of Internal Medicine, Erasmus Medical Center, 3015GE Rotterdam, The Netherlands

13. Analytic and Translational Genetics Unit, Massachusetts General Hospital and Harvard Medical School, Boston, MA, USA

14. MRC Epidemiology Unit, University of Cambridge, Institute of Metabolic Science, Addenbrooke’s Hospital, Hills Road, Cambridge, CB2 0QQ, UK

15. Institute of Social and Preventive Medicine (IUMSP), Centre Hospitalier Universitaire Vaudois (CHUV), Lausanne 1010, Switzerland

16. Swiss Institute of Bioinformatics, Lausanne 1015, Switzerland

17. Department of Medical Genetics, University of Lausanne, Lausanne 1005, Switzerland

18. Genetic Epidemiology Unit, Department of Epidemiology, Erasmus University Medical Center, 3015 GE Rotterdam, The Netherlands

19. Department of Genetics, University of North Carolina, Chapel Hill, NC 27599, USA

20. Channing Division of Network Medicine, Department of Medicine, Brigham and Women's Hospital and Harvard Medical School, Boston, MA 02115, USA

21. Division of Statistical Genomics, Department of Genetics Washington University School of Medicine, St. Louis, MO, USA

22. Department of Medical Epidemiology and Biostatistics, Karolinska Institutet, Stockholm 17177, Sweden

23. Department of Genetics, University Medical Center Groningen, University of Groningen, 9700 RB Groningen, The Netherlands

24. Wellcome Trust Centre for Human Genetics, University of Oxford, Oxford OX3 7BN, UK

25. Center for Statistical Genetics, Department of Biostatistics, University of Michigan, Ann Arbor, MI 48109, USA

26. Montreal Heart Institute, Montreal, Quebec H1T 1C8, Canada

27. Institute of Molecular and Cell Biology, University of Tartu, Tartu 51010, Estonia

28. Istituto di Ricerca Genetica e Biomedica (IRGB), Consiglio Nazionale delle Ricerche, Cagliari, Sardinia 09042, Italy

29. Wellcome Trust Sanger Institute, Hinxton, Cambridge CB10 1SA, UK

30. Institute for Medical Informatics, Biometry and Epidemiology (IMIBE), University Hospital Essen, Essen, Germany

31. Clinical Epidemiology, Integrated Research and Treatment Center, Center for Sepsis Control and Care (CSCC), Jena University Hospital, Jena, Germany

32. Department of Genetic Epidemiology, Institute of Epidemiology and Preventive Medicine, University of Regensburg, D-93053 Regensburg, Germany

33. Harvard School of Public Health, Department of Nutrition, Harvard University, Boston, MA 2115, USA

34. HudsonAlpha Institute for Biotechnology, Huntsville, AL 35806, USA

35. Institute of Genetic Epidemiology, Helmholtz Zentrum München - German Research Center for Environmental Health, D-85764 Neuherberg, Germany

36. Telethon Institute for Child Health Research, Centre for Child Health Research, The University of Western Australia, Western Australia 6008, Australia

37. Section on Growth and Development, Program in Developmental Endocrinology and Genetics, Eunice Kennedy Shriver National Institute of Child Health and Human Development, National Institutes of Health, Bethesda, MD 20892, USA

38. Netherlands Consortium for Healthy Aging (NCHA), Leiden University Medical Center, Leiden 2300 RC, The Netherlands

39. Department of Molecular Epidemiology, Leiden University Medical Center, 2300 RC Leiden, The Netherlands

40. Department of Human Genetics, Leiden University Medical Center, 2333 ZC Leiden, The Netherlands

41. Center for Complex Disease Genomics, McKusick-Nathans Institute of Genetic Medicine, Johns Hopkins University School of Medicine, Baltimore, MD 21205, USA

42. Cardiology, Department of Specialties of Internal Medicine, Geneva University Hospital, Geneva 1211, Switzerland

43. Department of Epidemiology Research, Statens Serum Institut, Copenhagen DK-2300, Denmark

44. Department of Genetics, Washington University School of Medicine, St. Louis, MO 63110, USA

45. Centre for Population Health Sciences, University of Edinburgh, Teviot Place, Edinburgh, EH8 9AG, Scotland, UK

46. Division of Cardiovacular Medicine, Radcliffe Department of Medicine, University of Oxford, Oxford OX3 9DU, UK

47. Division of Public Health Sciences, Fred Hutchinson Cancer Research Center, Seattle, WA 98109, USA

48. Department of Epidemiology, University of North Carolina at Chapel Hill, Chapel Hill, NC 27599, USA

49. William Harvey Research Institute, Barts and The London School of Medicine and Dentistry, Queen Mary University of London, EC1M 6BQ UK

50. Vth Department of Medicine (Nephrology, Hypertensiology, Endocrinology, Diabetology, Rheumatology), Medical Faculty of Mannheim, University of Heidelberg, Germany

51. Department of Internal Medicine II, Ulm University Medical Centre, D-89081 Ulm, Germany

52. National Institute for Health and Welfare, FI-00271 Helsinki, Finland

53. Epidemiology Program, University of Hawaii Cancer Center, Honolulu, HI USA

54. The Charles Bronfman Institute for Personalized Medicine, Icahn School of Medicine at Mount Sinai, New York, NY 10029, USA

55. Department of Twin Research and Genetic Epidemiology, King’s College London, London SE1 7EH, UK

56. Department of Cardiology, University Medical Center Groningen, University of Groningen, 9700RB Groningen, The Netherlands

57. Netherlands Consortium for Healthy Aging (NCHA), 3015GE Rotterdam, The Netherlands

58. Department of Epidemiology, Erasmus Medical Center, 3015GE Rotterdam, The Netherlands

59. Laboratory of Neurogenetics, National Institute on Aging, National Institutes of Health, Bethesda, MD 20892, USA

60. QIMR Berghofer Medical Research Institute, Queensland 4006, Australia

61. Oxford Centre for Diabetes, Endocrinology and Metabolism, University of Oxford, Oxford OX3 7LJ, UK

62. Department of Genomics of Common Disease, School of Public Health, Imperial College London, Hammersmith Hospital, London, UK

63. Stanley Center for Psychiatric Research, Broad Institute of MIT and Harvard, Cambridge, MA 02142, USA

64. Department of Clinical Sciences, Genetic & Molecular Epidemiology Unit, Lund University Diabetes Center, Skåne University Hosptial, Malmö 205 02, Sweden

65. Department of Public Health and Clinical Medicine, Unit of Medicine, Umeå University, Umeå 901 87, Sweden

66. Department of Odontology, Umeå University, Umeå 901 85, Sweden

67. University of Eastern Finland, FI-70210 Kuopio, Finland

68. Atherosclerosis Research Unit, Center for Molecular Medicine, Department of Medicine, Karolinska Institutet, Stockholm 17176, Sweden

69. Division of Biostatistics, Washington University School of Medicine, St. Louis, MO 63110, USA

70. Translational Gerontology Branch, National institute on Aging, Baltimore MD 21225, USA

71. Interfaculty Institute for Genetics and Functional Genomics, University Medicine Greifswald, D-17475 Greifswald, Germany

72. Department of Cardiology, Leiden University Medical Center, 2300 RC Leiden, The Netherlands

73. Department of Gerontology and Geriatrics, Leiden University Medical Center, 2300 RC Leiden, The Netherlands

74. Experimental Cardiology Laboratory, Division Heart and Lungs, University Medical Center Utrecht, 3584 CX Utrecht, The Netherlands

75. Department of Medical Genetics, University Medical Center Utrecht, 3584 CX Utrecht, The Netherlands

76. Department of Endocrinology, University of Groningen, University Medical Center Groningen, Groningen, 9700 RB, The Netherlands

77. DZHK (Deutsches Zentrum für Herz-Kreislaufforschung – German Centre for Cardiovascular Research), partner site Greifswald, D-17475 Greifswald, Germany

78. Institute of Clinical Chemistry and Laboratory Medicine, University Medicine Greifswald, D-17475 Greifswald, Germany

79. Division of Cancer Epidemiology and Genetics, National Cancer Institute, National Institutes of Health, Bethesda, MD 20892, USA

80. Core Genotyping Facility, SAIC-Frederick, Inc., NCI-Frederick, Frederick, MD 21702, USA

81. CNRS UMR 8199, F-59019 Lille, France

82. European Genomic Institute for Diabetes, F-59000 Lille, France

83. Université de Lille 2, F-59000 Lille, France

84. Ealing Hospital NHS Trust, Middlesex UB1 3HW, UK

85. Department of Epidemiology and Biostatistics, Imperial College London, London W2 1PG, UK

86. School of Health and Social Studies, Dalarna University, Falun, Sweden

87. PathWest Laboratory Medicine of Western Australia, NEDLANDS, Western Australia 6009, Australia

88. Geriatric Unit, Azienda Sanitaria Firenze (ASF), Florence, Italy

89. Department of Genetics, Texas Biomedical Research Institute, San Antonio, TX, USA

90. Department of Medical Sciences, Endocrinology, Diabetes and Metabolism, Uppsala University, Uppsala 75185, Sweden

91. IFB Adiposity Diseases, University of Leipzig, D-04103 Leipzig, Germany

92. Department of Medicine, University of Leipzig, D-04103 Leipzig, Germany

93. LifeLines, University Medical Center Groningen, University of Groningen, 9700 RB Groningen, The Netherlands

94. Department of Pharmacology and Therapeutics, University College Cork, Cork, Ireland

95. Department of Statistics & Biostatistics, Rutgers University, Piscataway, N.J. USA

96. Department of Genetics, Rutgers University, Piscataway, N.J. USA.

97. Department of Biology, Norwegian University of Science and Technology, Trondheim, Norway

98. Genome Technology Branch, National Human Genome Research Institute, NIH, Bethesda, MD 20892, USA

99. Clinical Trial Service Unit, Epidemiological Studies Unit, Nuffield Department of Population Health, University of Oxford, Oxford OX3 7LF, UK

100. Division of Endocrinology, Diabetes and Metabolism, Ulm University Medical Centre, D-89081 Ulm, Germany

101. Department of Radiology, University Medical Center Utrecht, Utrecht, The Netherlands

102. Department of Biomedical Informatics, Vanderbilt University, Nashville, TN 37232, USA

103. Department of Human Nutrition, Wageningen University, Wageningen, The Netherlands

104. Department of Dietetics-Nutrition, Harokopio University, Athens, Greece

105. Medical Research Institute, University of Dundee, Ninewells Hospital and Medical School, Dundee DD1 9SY, UK

106. Department of Internal Medicine B, University Medicine Greifswald, D-17475 Greifswald, Germany

107. Institute for Molecular Medicine, University of Helsinki, FI-00014 Helsinki, Finland

108. Laboratory of Epidemiology and Population Sciences, National Institute on Aging, NIH, Bethesda, MD 20892, USA

109. Department of Public Health and Caring Sciences, Geriatrics, Uppsala University, Uppsala 75185, Sweden

110. Kaiser Permanente, Division of Research, Oakland, CA 94612, USA

111. Research Unit of Molecular Epidemiology, Helmholtz Zentrum München - German Research Center for Environmental Health, D-85764 Neuherberg, Germany

112. German Center for Diabetes Research (DZD), Neuherberg, Germany

113. Department of Medicine III, University Hospital Carl Gustav Carus, Technische Universität Dresden, D-01307 Dresden, Germany

114. Department of Public Health and Clinical Medicine, Unit of Nutritional Research, Umeå University , Umeå 90187, Sweden

115. Department of Psychiatry, University of Groningen, University Medical Center Groningen, Groningen, The Netherlands

116. Kuopio Research Institute of Exercise Medicine, Kuopio, Finland

117. MRC Human Genetics Unit, Institute of Genetics and Molecular Medicine, University of Edinburgh, Western General Hospital, Edinburgh, EH4 2XU, Scotland, UK

118. National Heart, Lung, and Blood Institute, the Framingham Heart Study, Framingham MA 01702, USA

119. Department of Neurology, Boston University School of Medicine, Boston, MA 02118, USA

120. Department of Medical Statistics and Bioinformatics, Leiden University Medical Center, 2300 RC Leiden, The Netherlands

121. Faculty of Psychology and Education, VU University Amsterdam, Amsterdam, The Netherlands

122. Department of Epidemiology, University Medical Center Groningen, University of Groningen, 9700 RB Groningen, The Netherlands

123. Department of Medicine, Stanford University School of Medicine, Stanford, CA 94305, USA

124. Institute for Community Medicine, University Medicine Greifswald, D-17475 Greifswald, Germany

125. Division of Medical Genetics, Department of Biomedicine, University of Basel, Basel, Switzerland

126. Department of Genomics, Life & Brain Center, University of Bonn, Bonn, Germany

127. Institute of Human Genetics, University of Bonn, Bonn, Germany

128. Department of Public Health and General Practice, Norwegian University of Science and Technology, Trondheim 7489, Norway

129. Hannover Unified Biobank, Hannover Medical School, Hannover, D-30625 Hannover, Germany

130. Center for Medical Sytems Biology, Leiden, The Netherlands

131. Department of Pulmonary Physiology and Sleep Medicine, NEDLANDS, Western Australia 6009, Australia

132. School of Medicine and Pharmacology, University of Western Australia, CRAWLEY 6009, Australia

133. Uppsala University, Department of Immunology, Genetics & Pathology, SciLifeLab, Rudbeck Laboratory, SE-751 85, Uppsala, Sweden

134. Department of Haematology, University of Cambridge, Cambridge CB2 0PT, UK

135. NHS Blood and Transplant, Cambridge CB2 0PT, UK

136. Department of Medicine, University of Oulo, Oulo, Finland

137. Department of Medicine, Northwestern University Feinberg School of Medicine, Chicago, IL 60611, USA

138. Unit of Periodontology, Department of Restorative Dentistry, Periodontology and Endodontology, University Medicine Greifswald, D-17475 Greifswald, Germany

139. Department of Internal Medicine I, Ulm University Medical Centre, D-89081 Ulm, Germany

140. Institute of Human Genetics, Helmholtz Zentrum München - German Research Center for Environmental Health, D-85764 Neuherberg, Germany

141. Department of Medical Sciences, Cardiovascular Epidemiology, Uppsala University, Uppsala 75185, Sweden

142. Centre for Bone and Arthritis Research, Department of Internal Medicine and Clinical Nutrition, Institute of Medicine, Sahlgrenska Academy, University of Gothenburg, Gothenburg 413 45, Sweden

143. The Genetics of Obesity and Related Metabolic Traits Program, The Icahn School of Medicine at Mount Sinai, New York, NY 10029, USA

144. Steno Diabetes Center A, S, Gentofte DK-2820, Denmark

145. Service of Nephrology, Department of Medicine, Lausanne University Hospital (CHUV), Lausanne 1005, Switzerland 21

146. School of Social and Community Medicine, University of Bristol, Bristol BS8 2BN, UK

147. Tropical Metabolism Research Unit, Tropical Medicine Research Institute, The University of the West Indies, Mona, Kingston 7, Jamaica

148. Global Health Institute, Department of Life Sciences, École Polytechnique Fédérale de Lausanne, Lausanne, Switzerland

149. Institute of Microbiology, University Hospital and University of Lausanne, Lausanne 1011, Switzerland

150. The Center for Observational Research, Amgen, Inc., Thousand Oaks, CA 91320, USA

151. Center for Evidence-based Healthcare, University Hospital Carl Gustav Carus, Technische Universität Dresden, D-01307 Dresden, Germany

152. Department of Medicine I, University Hospital Grosshadern, Ludwig-Maximilians-Universität, D-81377 Munich, Germany

153. Institute of Medical Informatics, Biometry and Epidemiology, Chair of Genetic Epidemiology, Ludwig-Maximilians-Universität, D-85764 Neuherberg, Germany

154. Deutsches Forschungszentrum für Herz-Kreislauferkrankungen (DZHK) (German Research Centre for Cardiovascular Research), Munich Heart Alliance, D-80636 Munich, Germany

155. Department of Respiratory Medicine, Sir Charles Gairdner Hospital, NEDLANDS, Western Australia 6009, Australia

156. Department of Epidemiology and Biostatistics, EMGO Institute for Health and Care Research, VU University Medical Center, Amsterdam, The Netherlands

157. Department of Internal Medicine, Division of Endocrinology and Metabolism, Medical University of Graz, 8036 Graz, Austria

158. Diabetology-Endocrinology-Nutrition, AP-HP, Bichat Hospital, F-75018 Paris, France

159. INSERM, U872, Centre de Recherche des Cordeliers, F-75006 Paris, France

160. Paris Diderot University, F-75018 Paris, France

161. Clinical Institute of Medical and Chemical Laboratory Diagnostics, Medical University of Graz, Graz 8036, Austria

162. Department of Preventive Medicine, Keck School of Medicine, University of Southern California, Los Angeles, CA, USA

163. Deutsches Herzzentrum München, Technische Universität München, D-80636 Munich, Germany

164. National Cancer Institute, Bethesda, MD, USA

165. Department of Sociology, University of Helsinki, Helsinki FI-00014, Finland

166. EMGO Institute for Health and Care Research, VU University, 1081BT Amsterdam, The Netherlands

167. Department of Psychiatry, Neuroscience Campus, VU University Amsterdam, Amsterdam, The Netherlands

168. Icelandic Heart Association, Kopavogur 201, Iceland

169. University of Iceland, Reykjavik 101, Iceland

170. Molecular & Cellular Therapeutics, Royal College of Surgeons in Ireland, 123 St Stephens Green, Dublin 2, Ireland

171. Institute of Cardiovascular and Medical Sciences, Faculty of Medicine, University of Glasgow, Glasgow G12 8TA, UK

172. Department of Medical Sciences, Molecular Medicine, Uppsala University, Uppsala 75144, Sweden

173. Department of Public Health Sciences, Stritch School of Medicine, Loyola University of Chicago, Maywood, IL 61053, USA

174. deCODE Genetics, Amgen inc., Reykjavik 101, Iceland

175. Department of Ocology, University of Cambridge, Cambridge CB2 0QQ, UK

176. Department of Internal Medicine section of Geriatrics, Academic Medical Center, Amsterdam, The Netherlands

177. Department of Child and Adolescent Psychiatry, Psychology, Erasmus University Medical Centre, 3000 CB Rotterdam, The Netherlands

178. Department for Health Evidence, Radboud University Medical Centre, 6500 HB Nijmegen, The Netherlands

179. Department of Genetics, Radboud University Medical Centre, 6500 HB Nijmegen, The Netherlands

180. Department of Clinical Chemistry, Ulm University Medical Centre, D-89081 Ulm, Germany

181. Deutsches Forschungszentrum für Herz-Kreislauferkrankungen (DZHK) (German Research Centre for Cardiovascular Research), partner site Hamburg/Lubeck/Kiel, Lubeck, Germany

182. Institut für Integrative und Experimentelle Genomik, Universität zu Lübeck, D-23562 Lübeck, Germany

183. Department of Community Medicine, Faculty of Health Sciences, UiT The Arctic University of Tromsø, Tromsø, Norway

184. MRC Unit for Lifelong Health and Ageing at UCL, London WC1B 5JU, UK

185. Department of Epidemiology and Public Health, EA3430, University of Strasbourg, Faculty of Medicine, Strasbourg, France

186. Department of Internal Medicine, University Medical Center Groningen, University of Groningen, 9700RB Groningen, The Netherlands

187. Pathology and Laboratory Medicine, The University of Western Australia, Western Australia 6009, Australia

188. Cedars-Sinai Diabetes and Obesity Research Institute, Los Angeles, CA, USA

189. Department of Prosthetic Dentistry, Gerostomatology and Dental Materials, University Medicine Greifswald, D-17475 Greifswald, Germany

190. Biological Psychology, VU University Amsterdam, 1081BT Amsterdam, The Netherlands

191. Institute of Social and Preventive Medicine (IUMSP), Centre Hospitalier Universitaire Vaudois and University of Lausanne, Lausanne, Switzerland

192. Ministry of Health, Victoria, Republic of Seychelles

193. Laboratory Medicine, Hospital of Desio, department of Health Sciences, University of Milano, Bicocca, Italy

194. Clinical Pharmacology Unit, University of Cambridge, Addenbrooke's Hospital, Hills Road, Cambridge CB2 2QQ, UK

195. Clinical Pharmacology and Barts and The London Genome Centre, William Harvey Research Institute, Barts and The London School of Medicine and Dentistry, Queen Mary University of London, Charterhouse Square, London EC1M 6BQ, UK

196. Center for Human Genetics Research, Vanderbilt University Medical Center, Nashville TN 37203, USA

197. Department of Molecular Physiology and Biophysics, Vanderbilt University, Nashville, TN 37232, USA

198. Department of Biostatistics, Boston University School of Public Health, Boston, MA 02118, USA

199. Department of Public Health and Primary Care, University of Cambridge, Cambridge, UK

200. Division of Cardiovascular Epidemiology, Institute of Environmental Medicine, Karolinska Institutet, Stockholm, Sweden, Stockholm 17177, Sweden

201. Julius Center for Health Sciences and Primary Care, University Medical Center Utrecht, 3584 CX Utrecht, The Netherlands

202. Clinic of Cardiology, West-German Heart Centre, University Hospital Essen, Essen, Germany

203. Department of General Practice and Primary Health Care, University of Helsinki, FI-00290 Helsinki, Finland

204. Unit of General Practice, Helsinki University Central Hospital, Helsinki 00290, Finland

205. Department of Internal Medicine, University of Pisa, Pisa, Italy

206. CNR Institute of Clinical Physiology, University of Pisa, Pisa, Italy

207. Department of Cardiology, Toulouse University School of Medicine, Rangueil Hospital, Toulouse, France

208. Robertson Center for Biostatistics, University of Glasgow, Glasgow, UK

209. NorthShore University HealthSystem, Evanston, IL, University of Chicago, Chicago, IL, USA

210. Service of Therapeutic Education for Diabetes, Obesity and Chronic Diseases, Geneva University Hospital, Geneva CH-1211, Switzerland

211. Vanderbilt University School of Medicine, Department of Medicine, Pharmacology, Pathology, Microbiology and Immunology, Nashville, Tennessee, USA

212. Leeds MRC Medical Bioinformatics Centre, University of Leeds, UK

213. Institute of Biomedical & Clinical Science, University of Exeter, Barrack Road, Exeter, EX2 5DW

214. Department of Psychiatry, Washington University School of Medicine, St. Louis, MO 63110, USA

215. Center for Biomedicine, European Academy Bozen, Bolzano (EURAC), Bolzano 39100, Italy

216. Affiliated Institute of the University of Lübeck, D-23562 Lübeck, Germany

217. Division of Genomic Medicine, National Human Genome Research Institute, National Institutes of Health, Bethesda, MD, USA

218. Institute of Cardiovascular Science, University College London, WC1E 6BT, UK

219. Department of Vascular Medicine, Academic Medical Center, Amsterdam, The Netherlands

220. Centre for Cardiovascular Genetics, Institute Cardiovascular Sciences, University College London, London WC1E 6JJ, UK

221. Cardiovascular Genetics Division, Department of Internal Medicine, University of Utah, Salt Lake City, Utah 84108, USA

222. School of Population Health and Sansom Institute for Health Research, University of South Australia, Adelaide 5000, Australia

223. South Australian Health and Medical Research Institute, Adelaide, Australia

224. Centre for Paediatric Epidemiology and Biostatistics, UCL Institute of Child Health, London WC1N 1EH, UK

225. National Institute for Health and Welfare, FI-90101 Oulu, Finland

226. MRC Health Protection Agency (HPE) Centre for Environment and Health, School of Public Health, Imperial College London, UK

227. Unit of Primary Care, Oulu University Hospital, FI-90220 Oulu, Finland

228. Biocenter Oulu, University of Oulu, FI-90014 Oulu, Finland

229. Institute of Health Sciences, FI-90014 University of Oulu, Finland

230. Hjelt Institute Department of Public Health, University of Helsinki, FI-00014 Helsinki, Finland

231. Department of Forensic Molecular Biology, Erasmus MC, 3015GE Rotterdam, The Netherlands

232. UKCRC Centre of Excellence for Public Health (NI), Queens University of Belfast, Northern Ireland

233. Faculty of Medicine, Institute of Health Sciences, University of Oulu, Oulu, Finland

234. Unit of General Practice, Oulu University Hospital, Oulu, Finland

235. Department of Urology, Radboud University Medical Centre, 6500 HB Nijmegen, The Netherlands

236. Imperial College Healthcare NHS Trust, London W12 0HS, UK

237. National Heart and Lung Institute, Imperial College, London W12 0NN, UK

238. Department of Epidemiology and Public Health, UCL London, WC1E 6BT, UK

239. Department of Medicine, Kuopio University Hospital and University of Eastern Finland, FI-70210 Kuopio, Finland

240. Department of Physiology, Institute of Biomedicine, University of Eastern Finland, Kuopio Campus, Kuopio, Finland

241. Department of Clinical Physiology and Nuclear Medicine, Kuopio University Hospital and University of Eastern Finland, Kuopio, Finland

242. Department of Clinical Chemistry, Fimlab Laboratories and School of Medicine University of Tampere, FI-33520 Tampere, Finland

243. Department of Health Sciences, University of Milano, I 20142, Italy

244. Fondazione Filarete, Milano I 20139, Italy

245. Division of Nephrology and Dialysis, San Raffaele Scientific Institute, Milano I 20132, Italy

246. Università Vita-Salute San Raffaele, Milano I 20132, Italy

247. Institut Universitaire de Cardiologie et de Pneumologie de Québec, Faculty of Medicine, Laval University, Quebec, QC G1V 0A6, Canada

248. Institute of Nutrition and Functional Foods, Laval University, Quebec, QC G1V 0A6, Canada

249. Department of Biostatistics, University of Washington, Seattle, WA 98195, USA

250. Department of Surgery, University Medical Center Utrecht, 3584 CX Utrecht, The Netherlands

251. Department of Biostatistics, University of Liverpool, Liverpool L69 3GA, UK

252. Department of Pediatrics, University of Iowa, Iowa City, Iowa IA 52242, USA

253. Institute of Epidemiology II, Helmholtz Zentrum München - German Research Center for Environmental Health, Neuherberg, Germany, D-85764 Neuherberg, Germany

254. Department of Neurology, General Central Hospital, Bolzano 39100, Italy

255. Department of Nutrition, Harvard School of Public Health, Boston, Massachusetts, USA

256. Department of Clinical Physiology and Nuclear Medicine, Turku University Hospital, FI-20521 Turku, Finland

257. Research Centre of Applied and Preventive Cardiovascular Medicine, University of Turku, FI-20521 Turku, Finland

258. Human Genomics Laboratory, Pennington Biomedical Research Center, Baton Rouge, LA 70808, USA

259. Center for Systems Genomics, The Pennsylvania State University, University Park, PA 16802, USA

260. Croatian Centre for Global Health, Faculty of Medicine, University of Split, 21000 Split, Croatia

261. Department of Cardiovascular Sciences, University of Leicester, Glenfield Hospital, Leicester LE3 9QP, UK

262. National Institute for Health Research (NIHR) Leicester Cardiovascular Biomedical Research Unit, Glenfield Hospital, Leicester, LE3 9QP, UK

263. South Carelia Central Hospital. 53130 Lappeenranta. Finland

264. Paul Langerhans Institute Dresden, German Center for Diabetes Research (DZD), Dresden, Germany

265. International Centre for Circulatory Health, Imperial College London, London W2 1PG, UK

266. Program for Personalized and Genomic Medicine, and Division of Endocrinology, Diabetes and Nutrition, University of Maryland School of Medicine, Baltimore, MD 21201, USA

267. Geriatric Research and Education Clinical Center, Vetrans Administration Medical Center, Baltimore, MD 21201, USA

268. HUCH Heart and Lungcenter, Department of Medicine, Helsinki University Central Hospital, FI-00290 Helsinki, Finland

269. Université de Montréal, Montreal, Quebec H1T 1C8, Canada

270. Department of Kinesiology, Laval University, Quebec, QC G1V 0A6, Canada

271. Dipartimento di Scienze Farmacologiche e Biomolecolari, Università di Milano & Centro Cardiologico Monzino, IRCCS, Milan 20133, italy

272. Department of Food Science and Nutrition, Laval University, Quebec, QC G1V 0A6, Canada

273. The electronic medical records and genomics (eMERGE) consortium

274. Myocardial Infarction Genetics (MIGen) Consortium

275. Membership to this consortium is provided below.

276. Population Architecture using Genomics and Epidemiology Consortium

277. The LifeLines Cohort Study, University of Groningen, University Medical Center Groningen, Groningen, The Netherlands

278. Institut Pasteur de Lille; INSERM, U744; Université de Lille 2; F-59000 Lille, France

279. Department of Cardiology, Division Heart and Lungs, University Medical Center Utrecht, 3584 CX Utrecht, The Netherlands

280. Durrer Center for Cardiogenetic Research, Interuniversity Cardiology Institute Netherlands-Netherlands Heart Institute, 3501 DG Utrecht, The Netherlands

281. Lee Kong Chian School of Medicine, Imperial College London and Nanyang Technological University, Singapore, 637553 Singapore, Singapore

282. Health Science Center at Houston, University of Texas, Houston, TX, USA

283. Department of Medicine, Division of Genetics, Brigham and Women's Hospital, Harvard Medical School, Boston, MA 02115, USA

284. Department of Epidemiology, University Medical Center Utrecht, Utrecht, The Netherlands

285. Lund University Diabetes Centre and Department of Clinical Science, Diabetes & Endocrinology Unit, Lund University, Malmö 221 00, Sweden

286. Harvard School of Public Health, Department of Epidemiology, Harvard University, Boston, MA 2115, USA

287. Interuniversity Cardiology Institute of the Netherlands (ICIN), Utrecht, the Netherlands

288. Albert Einstein College of Medicine. Department of epidemiology and population health, Belfer 1306, NY 10461, USA

289. Center for Human Genetics, Division of Public Health Sciences, Wake Forest School of Medicine, Winston-Salem, NC 27157, USA

290. Synlab Academy, Synlab Services GmbH, Mannheim, Germany

291. Department of Clinical Genetics, Erasmus University Medical Center, Rotterdam, The Netherlands

292. Harvard Medical School, Boston, MA 02115, USA

293. Institute for Translational Genomics and Population Sciences, Los Angeles BioMedical Research Institute at Harbor-UCLA Medical Center, Torrance, CA, USA

294. Finnish Diabetes Association, Kirjoniementie 15, FI-33680 Tampere, Finland

295. Pirkanmaa Hospital District, Tampere, Finland

296. Center for Non-Communicable Diseases, Karatchi, Pakistan

297. Department of Medicine, University of Pennsylvania, Philadelphia, USA

298. Laboratory of Genetics, National Institute on Aging, Baltimore, MD 21224, USA

299. Instituto de Investigacion Sanitaria del Hospital Universario LaPaz (IdiPAZ), Madrid, Spain

300. Diabetes Research Group, King Abdulaziz University, Jeddah, Saudi Arabia

301. Centre for Vascular Prevention, Danube-University Krems, 3500 Krems, Austria

302. Department of Public Health and Clinical Nutrition, University of Eastern Finland, Finland

303. Research Unit, Kuopio University Hospital, Kuopio, Finland

304. Institute of Cellular Medicine, Newcastle University, Newcastle NE1 7RU, UK

305. Institute of Medical Informatics, Biometry and Epidemiology, Chair of Epidemiology, Ludwig-Maximilians-Universität, D-85764 Munich, Germany

306. Klinikum Grosshadern, D-81377 Munich, Germany

307. Institute of Epidemiology I, Helmholtz Zentrum München - German Research Center for Environmental Health, Neuherberg, Germany, D-85764 Neuherberg, Germany

308. Department of Pulmonology, University Medical Center Utrecht, Utrecht, The Netherlands

309. King Abdulaziz University, Jeddah 21589, Saudi Arabia

310. Department of Internal Medicine, Division of Gastroenterology, and Department of Computational Medicine and Bioinformatics, University of Michigan, Ann Arbor, MI 48109

311. Faculty of Medicine, University of Iceland, Reykjavik 101, Iceland

312. University of Cambridge Metabolic Research Laboratories, Institute of Metabolic Science, Addenbrooke’s Hospital, Cambridge CB2 OQQ, UK

313. NIHR Cambridge Biomedical Research Centre, Institute of Metabolic Science, Addenbrooke’s Hospital, Cambridge CB2 OQQ, UK

314. Carolina Center for Genome Sciences, University of North Carolina at Chapel Hill, Chapel Hill, NC 27599, USA

315. Division of Population Health Sciences & Education, St George's, University of London, London SW17 0RE, UK

316. Service of Medical Genetics, CHUV University Hospital, Lausanne, Switzerland

317. Oxford NIHR Biomedical Research Centre, Oxford University Hospitals NHS Trust, Oxford, OX3 7LJ, UK

318. Department of Internal Medicine, Division of Cardiovascular Medicine, University of Michigan, Ann Arbor, MI, USA

319. Department of Computational Medicine and Bioinformatics, University of Michigan, Ann Arbor, MI, USA

320. Department of Human Genetics, University of Michigan, Ann Arbor, MI, USA

321. Harvard School of Public Health, Department of Biostatistics, Boston, MA 02115, USA

322. The Mindich Child Health and Development Institute, Icahn School of Medicine at Mount Sinai, New York, NY 10029, USA

323. Biosciences Research Division, Department of Primary Industries, Victoria 3083, Australia

324. Department of Food and Agricultural Systems, University of Melbourne, Victoria 3010, Australia
